# AptaMat: a matrix-based algorithm to compare single-stranded oligonucleotides secondary structures

**DOI:** 10.1093/bioinformatics/btac752

**Published:** 2022-11-28

**Authors:** Thomas Binet, Bérangère Avalle, Miraine Dávila Felipe, Irene Maffucci

**Affiliations:** Université de technologie de Compiègne, UPJV, CNRS, Enzyme and Cell Engineering, Centre de recherche Royallieu, CS 60 319 - 60 203, Compiègne Cedex, France; Université de technologie de Compiègne, UPJV, CNRS, Enzyme and Cell Engineering, Centre de recherche Royallieu, CS 60 319 - 60 203, Compiègne Cedex, France; Université de technologie de Compiègne, LMAC (Laboratory of Applied Mathematics of Compiègne), CS 60 319 - 60 203, Compiègne Cedex, France; Université de technologie de Compiègne, UPJV, CNRS, Enzyme and Cell Engineering, Centre de recherche Royallieu, CS 60 319 - 60 203, Compiègne Cedex, France

## Abstract

**Motivation:**

Comparing single-stranded nucleic acids (ssNAs) secondary structures is fundamental when investigating their function and evolution and predicting the effect of mutations on their structures. Many comparison metrics exist, although they are either too elaborate or not sensitive enough to distinguish close ssNAs structures.

**Results:**

In this context, we developed AptaMat, a simple and sensitive algorithm for ssNAs secondary structures comparison based on matrices representing the ssNAs secondary structures and a metric built upon the Manhattan distance in the plane. We applied AptaMat to several examples and compared the results to those obtained by the most frequently used metrics, namely the Hamming distance and the RNAdistance, and by a recently developed image-based approach. We showed that AptaMat is able to discriminate between similar sequences, outperforming all the other here considered metrics. In addition, we showed that AptaMat was able to correctly classify 14 RFAM families within a clustering procedure.

**Availability and implementation:**

The python code for AptaMat is available at https://github.com/GEC-git/AptaMat.git.

**Supplementary information:**

Supplementary data are available at *Bioinformatics* online.

## 1 Introduction

Single-stranded nucleic acids (ssNAs) are interesting molecules from both a biological and a biotechnological point of view. On one side, RNA is fundamental for protein synthesis, and it has cellular structural, functional and regulatory roles. On the other side, both RNA and single-stranded DNA, in the form of aptamers, can be exploited as therapeutic or diagnostic tools or as biosensors (Kulabhusan *et al.*, 2020). Aptamers are, indeed, short single-stranded oligonucleotides able to bind a large variety of molecular targets with high specificity and dissociation constants in the nano- to picomolar range by adopting specific conformations (Li *et al.*, 2021; Nimjee *et al.*, 2017).

**Fig. 1.**
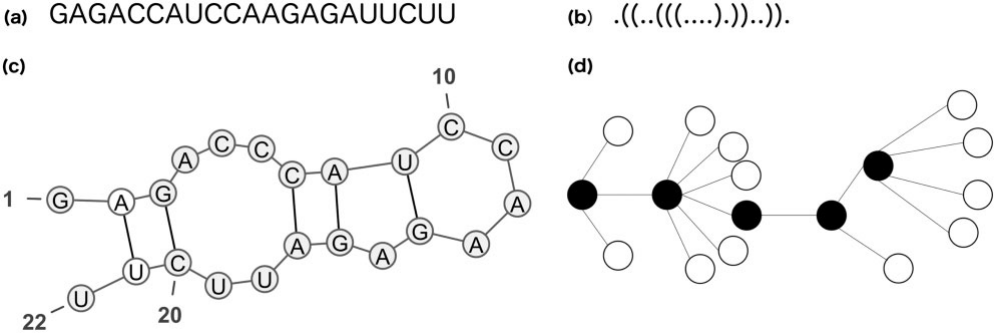
Example of representations of the secondary structure of sequence (**a**): dot-bracket notation (**b**), graphical representation realized with VARNA (Darty *et al.*, 2009) (**c**) and full tree representation (**d**)

SsNAs function highly depends on their secondary (i.e. their base pairing pattern) and tertiary (i.e. their 3D organization) structures (Li *et al.*, 2021; Mustoe *et al.*, 2014; Nimjee *et al.*, 2017), thus the computational prediction of these two levels of organization can help to understand ssNAs roles and interactions with other molecules. The prediction of the ssNAs secondary structures often precedes and guides the 3D modeling step and many tools have been developed at this scope (Gruber *et al.*, 2008b; Sato *et al.*, 2009; Zuker, 2003). The resulting output is usually a graphical representation of the predicted secondary structure (Fig. 1c) and/or its dot-bracket notation (Fig. 1b), which consists in a string of the same length as the sequence based on an alphabet of three characters: {‘.’, ‘(“,”)’}. The symbol ‘.’ indicates that the nucleotide in the corresponding position is unpaired, while ‘(“and”)’ correspond to the opening and closing positions of a base pair (BP), respectively.

The comparison of ssNAs secondary structures is a task of considerable importance. Comparing ssNAs structures can help to study the function and evolution of ssNAs, to design nucleotide sequences that fold into a given secondary structure, facing the task of inverse folding, in order to optimize ssNAs properties, but also to guide the computational detection of new non-coding RNAs (Churkin *et al.*, 2018). In addition, ssNAs structures comparison can assist the prediction of mutations that can cause a conformational rearrangement (Barash and Churkin, 2011). Therefore, different algorithms have been developed at this scope (see Gruber *et al.* (2008a) for a review). Briefly, these can be classified in algorithms (i) based on the minimum free energy (Washietl *et al.*, 2005), (ii) based on a single structure (Flamm *et al.*, 2001; Fontana *et al.*, 1993; Moulton *et al.*, 2000; Shapiro *et al.*, 1988) and (iii) considering the whole folding space (Bonhoeffer *et al.*, 1993; Giegerich *et al.*, 2004; Hofacker *et al.*, 1994). Among them, the most frequently applied are those working on single structures, such as the Hamming distance (Hamming, 1950), the BP distance and the RNAdistance algorithm implemented in the Vienna package (Hofacker, 2003). The Hamming distance allows the comparison of two strings of the same length by counting the number of positions with different symbols. It is one of the simplest metrics used in the context of ssNAs, and it is usually calculated by counting the number of positions with different nucleotides (Equation 3). It can be adapted to strings in the dot-bracket notation, which is more suitable for secondary structures comparison. Conversely, the default RNAdistance implemented in the Vienna package is based on the comparison of ssNAs secondary structures represented as ordered rooted trees in a full resolution (Fig. 1d) (Gruber *et al.*, 2008b). Besides this default approach, the Vienna package offers the possibility to compare ssNA structures by either a rooted tree editing comparison of homeomorphically irreducible trees (HIT), weighted coarse-grained trees and coarse-grained trees or by a comparison of the HIT, coarse-grained or weighted coarse-grained structure strings. In addition, the Vienna RNAdistance can also compute the BP distance, which counts the total number of BPs that occur in one of the structures, but not in the other one (Gruber *et al.*, 2008b).

**Fig. 2.**
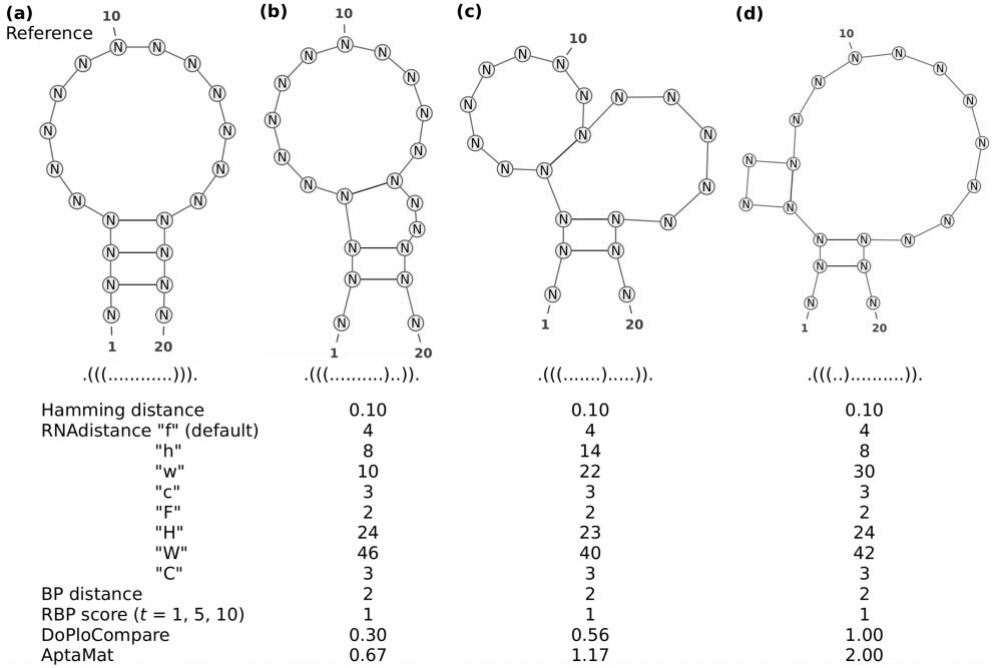
Reference (**a**) and alternative (**b**, **c** and **d**) structures for ssNA 1. The Hamming, RNAdistance with the different options, BP, DoPloCompare, AptaMat distances and the RBP scores (with *t* = 1, 5 and 10) are computed using structure (a) as reference

However, all the previously cited metrics sometimes fail in finding differences between secondary structures as shown in the example of Figure 2 adapted from Ivry *et al.* (2009), where the Hamming distance, the default RNAdistance (‘f’), and the BP distance cannot capture the differences between structures (b), (c) or (d) and the reference structure (a). Indeed, the Hamming distance only considers the total number of matching positions, without taking into account the correlations between the opening and closing positions, which are characteristic for the structure. Similarly, the BP distance counts the number of mismatching BPs, which might be the same, although the structures clearly have different distances from a reference, as in Figure 2. On the other hand, RNAdistance works with a tree representation that, even at full resolution (i.e. without any loss of information with regard to the dot-bracket notation), might lead to an equivalent cost in the tree editing operations for structures that seem to have a different degree of proximity to the reference one. This is illustrated in Figure 2, and the details about the computation of RNAdistance can be found in Supplementary Figure S1. In addition, the RNAdistance between two structures is highly dependent on the chosen computing option, as it can be seen when analyzing the structures of Figure 2.

Efforts have been done to solve the sensitivity issue of the above-mentioned metrics. For example, a relaxed BP (RBP) score has been developed to increase the ability of the BP distance in comparing ssNAs secondary structures (Agius *et al.*, 2010) by introducing a relaxation parameter. However, the greater flexibility of the RBP score as compared to the standard BP distance might not be enough to capture differences between the structures as shown in the example in Figure 2.

Other interesting approaches based on image processing, such as DoPloCompare (Ivry *et al.*, 2009), have been developed with a similar goal. These approaches consist in representing the secondary structures of the two compared ssNAs as dotplots and then processing them as images in order to measure the distance between the two structures. The use of dotplots allows taking into account the BPs’ relative positions and it provides a finer description of the ssNA structure than RNAdistance (Ivry *et al.*, 2009). However, this approach can be laborious and sometimes it fails in finding the expected trend when comparing multiple structures to a reference one, as we will see later. Indeed, although the image processing approach is a novelty in the field, the proposed metrics use a combination of geometrical distance and histogram correlations that might hinder the nature of the proximity between the compared structures. Moreover, DoPloCompare seems to be not symmetric, which is an important requirement for many applications.

Although there exist several other approaches to compare secondary structures, to our knowledge, none of them satisfy the desired properties: (i) simple in terms of results interpretation; (ii) easy to implement and to manipulate; (iii) exploitable for the comparison of pairs of structures, but also of multiple structures to a reference one, and, most of all and (iv) sensitive, in order to properly differentiate particularly close structures. Therefore, we developed a new algorithm, called AptaMat, which solves the issues of both the single structure-based and the image-based approaches. Briefly, AptaMat takes as input the aligned secondary structures of two ssNAs (*S_A_* and *S_B_*) of length *L* in the dot-bracket notation and creates for each of them a matrix of size *L *×* L*, comparable to a dotplot with 1 and 0 instead of dots and blank cells, respectively. Indeed, the (*i*,*j*)th entry of the matrix is either equal to 1 if the nucleotide in position *i* is paired with the nucleotide in position *j* or 0 if the nucleotides in positions *i* and *j* are not paired or in presence of a gap. For each BP of each structure, we find the closest BP on the other structure using the Manhattan distance between points in the plane. The distances between all the closest pairs are summed up, the number of introduced gaps is added and the resulting sum is normalized by the total number of cells containing 1 in both matrices, in order to find the final AptaMat distance (Supplementary Figs S2 and S3). A weighted AptaMat distance can also be computed to consider simultaneously multiple alternative conformations.

We applied our approach to (i) five examples taken from the work by Ivry *et al.* (2009) to make a direct comparison with the Hamming distance, RNAdistance and DoPloCompare and (ii) to five structures of aptamers taken from the Protein Data Bank (Berman *et al.*, 2000). The obtained results show that AptaMat is able to properly compare ssNAs secondary structures and to well discriminate among different structures. Finally, AptaMat also showed a good performance in clustering RNA structures according to their belonging RFAM families.

The python code implementing AptaMat is available on GitHub at https://github.com/GEC-git/AptaMat.git.

## 2 Materials and methods

### 2.1 AptaMat algorithm

The AptaMat algorithm has been developed for the comparison and quantification of the differences between aligned structures of pairs of ssNAs, with the alignment being of length *L*.

The algorithm takes as input the two pairwise aligned structures written in the dot-bracket notation, with one structure considered as reference. Starting from each input dot-bracket string a square matrix of *L *×* L* in size is created, where each matrix cell (*i*, *j*) corresponds to the position *i* of a nucleotide of the sequence relative to another position *j* of the same sequence. Therefore, each cell (*i*, *j*) contains either 1, if the nucleotide in position *i* is involved in a BP with the nucleotide in position *j*, or 0 if not. Cells corresponding to positions with gaps contain 0 as well. The resulting matrices can be assimilated to dotplots, with 1 instead of a dot and 0 instead of blank cells. Although very simple, this representation allows taking into account the relative position of the BPs in the ssNA sequence, thus retaining a more complete structural information as compared to the dot-bracket notation. The python implementation of AptaMat allows performing the structures alignment internally, by calling the RNAlign2D (Woźniak *et al.*, 2021) structure alignment tool. Alternatively, the user can provide his/her own alignment.

For the clarity of the algorithm description, we will call matrix $A=(a_{ij})$ the one containing the information regarding the reference structure and matrix $B=(b_{ij})$ the one storing the information of the structure we want to compare to the reference one. We want to define a distance between these matrices that reflects the proximity between cells containing 1 in both of them, i.e. those indicating a BP. For this purpose, each matrix is embedded in the plane in the following way: each (*i, j*)th entry that is equal to 1 is assimilated to the point with coordinates (j,L−i+1). Hence, to a matrix representing a secondary structure we associate a set of points in the plane with coordinates in ${\left\{1,\ldots ,L\right\}}^{2}$. Moreover, since both matrices are symmetrical, we consider only the entries below the diagonal. More precisely, let $P_{A}:=\{(j,L-i+1)\in N^{2}:a_{ij}=1,\;1\leq j<i\leq L\}$ be the set of points corresponding with structure *S_A_*. The set $P_{B}$ is defined analogously. A natural way to measure the distance between the BPs in the compared structures is to measure the distance between sets $P_{A}$ and $P_{B}$. At this scope, any distance between compact sets of points in $R^{2}$ could be appropriate for the method (e.g. Haussdorf distance, Huttenlocher *et al.*, 1993). At the moment, AptaMat algorithm implements a metric based on the Manhattan distance, which was chosen for its simplicity, as it is expressed as the sum of the absolute differences between the coordinates of the compared points (Krause, 1988). However, other distances can be easily implemented.

In AptaMat, for each point *P* in $P_{A}$ we find the Manhattan distance to its nearest neighbor in $P_{B}$, and vice versa. In order to handle all the differences between the structures, it is important to consider the distance in both directions (Supplementary Figs S2 and S3). Indeed, both structures do not have necessarily the same number of BPs. As a consequence, the distances in the two directions might not be the same and, more importantly, some BPs might be excluded from the comparison. Therefore, considering only the distances in one direction might be source of mistakes. Then, the shortest distances between $P_{A}$ and $P_{B}$ sets are summed up. The insertion of gaps within the alignment will determine the increase of the distance between two points, therefore we introduced a gap cost of 1 as indicated in Eq. 1. Finally, the obtained distance is normalized by the total number of BPs in structures *S_A_* and *S_B_*, since some distances might emerge twice in the calculation. Notice that this sort of normalization gives a more important weight to BPs in common between the two compared structures. The AptaMat distance, denoted by $D_{\text{AM}}$ is, therefore, defined as

(1) $$D_{\text{AM}}\left(S_{A},S_{B}\right)=\frac{\sum_{P\in P_{A}}d_{\text{Man}}\left(P,P_{B}\right)+\sum_{P\in P_{B}}d_{\text{Man}}\left(P,P_{A}\right)+N_{G}}{\#P_{A}+\#P_{B}},$$

where, for any given point $P=(x,y)\in R^{2}$ and any finite subset $C\subset R^{2}$, we denote by #C the cardinal of C, and by $d_{\text{Man}}(P,C)$ the Manhattan distance from *P* to its nearest neighbor in C. Finally, *N_G_* denotes the number of gaps in the alignment.

We can easily check that $D_{\text{AM}}$ is symmetric, and it is equal to 0 only when both structures are identical. In the light of this, the more the AptaMat distance is close to 0 the more the two compared structures are similar, independently of their length.

Because of their intrinsic flexibility, ssNAs can experimentally adopt many conformations, leading to an ensemble of structures (Ganser *et al.*, 2019). To take this into account, AptaMat, with the option ‘ – *ensemble*’, allows taking as input an ensemble of *n* structures ${\left(B_{i}\right)}_{i=1}^{n}$ and their associated weights ${\left(w_{i}\right)}_{i=1}^{n}$, which can be derived from either experimental data or prediction tools. In this case, the weighted AptaMat distance is calculated as

(2) $$D_{\text{AM}}\left(S_{A},{\left(S_{B_{i}}\right)}_{i=1}^{n}\right)=\sum_{i=1}^{n}w_{i}D_{\text{AM}}(S_{A},S_{B_{i}}).$$

### 2.2 Test set preparation

In order to confront AptaMat to the most used metrics for ssNAs comparison, we built a test set of 10 ssNA with known structures: five taken from the work by Ivry *et al.* (2009) and five taken from the PDB database (Supplementary Table S1). The selected ssNA have different lengths (20–127 nucleotides) and different secondary structures, containing stems, hairpin/stem loops, bulges, internal loops and junctions. For each sequence, the reference secondary structure in the dot-bracket notation was either taken from Ivry *et al.* (2009) or extrapolated using ×3dna-dssr (Lu and Olson, 2003) and then used as the reference structure. In addition, for each sequence, two or more alternative structures were used to perform the comparison. The alternative structures for the examples taken from Ivry *et al.* (2009) were obtained from the same article, while for those taken from the PDB database we used six different ssNA secondary structure prediction tools, namely Mfold (Zuker, 2003), LinearFold (Huang *et al.*, 2019), CentroidFold (Hamada *et al.*, 2009), RNAfold (Gruber *et al.*, 2008b), RNAstructure (Reuter and Mathews, 2010) and MC-Fold (Parisien and Major, 2008) to obtain at least two different secondary structures for each ssNA. This was achieved when the prediction tools were not able to correctly predict the secondary structure of the processed sequences.

### 2.3 Comparison methods

We compared AptaMat to some of the most used methods of ssNAs secondary structures comparison: the Hamming distance (Hamming, 1950), the BP distance and RNAdistance from the ViennaRNA package (Hofacker, 2003). The first computes the distance between two ssNAs structures of same length *L*, by calculating

(3) $$D_{\text{Hamming}}\left(S_{A},S_{B}\right)=N_{\text{diff}}/L,$$

where $N_{\text{diff}}$ is the number of unmatched positions between the two strings corresponding to the dot-bracket notation of the compared structures.

In order to compute the BP distance, which counts the total number of BPs occurring in one structure but not in the other, we used the option ‘P’ of the Vienna RNAdistance tool. In addition, when the BP distance could not capture the differences between the considered structures, we also computed the RBP distance as described in Agius *et al.* (2010) with *t* set to 1, 5 and 10, since the authors suggest that *t* values between 0.05 and 20 provide good results.

The Vienna implementation of RNAdistance allows computing the distance between two ssNA structures in multiple ways depending on the chosen option. The default RNAdistance implemented in the Vienna package (parameter ‘f’) computes the distance between two ssNAs structures by representing them as ordered rooted trees. At full resolution, this representation is deducible from the dot-bracket notation by assigning each unpaired nucleotide to a leaf and each BP to an internal node, as shown in Figure 1d. In order to calculate the distance between two trees, the tree editing approach is used, which consists in a series of edit operations (deletion, insertion or mutation of a node), to which a cost is assigned and that allow to transform a tree *T_A_* into a tree *T_B_*. The resulting distance $D_{\text{RNA}}(S_{A},S_{B})$ corresponds to the minimal total cost of the series of operations allowing to transform one tree into the other. The tree editing comparison is also used when parameters ‘h’, ‘w’ and ‘c’ are chosen, i.e. when the ssNAs structures are represented as HIT, weighted coarse-grained or coarse-grained trees, respectively. As an alternative to the tree editing comparison, Vienna RNAdistance offers the possibility to make a string comparison after the conversion of the dotbracket structure to a string indicating structural elements, such as paired or unpaired bases, or hairpins and bulges, depending on the type of the coarse grain representation (parameters ‘F’, ‘H’, ‘W’ and ‘C’).

In addition, for the structures taken from Ivry *et al.* (2009) (Supplementary Table S1), we included in the benchmark of AptaMat the comparison with the algorithm DoPloCompare, which uses an approach based on image processing to measure the distance between two ssNAs secondary structures. This algorithm has been selected for comparison with AptaMat because of its higher sensitivity as compared to the Hamming distance and RNAdistance (Fig. 2), and because it is based on the dotplot diagrams of the compared structures, as AptaMat. The distance grade proposed in this algorithm to compare two structures *S_A_* and *S_B_* can be defined as

(4) $$D_{\text{DoPloCompare}}\left(S_{A},S_{B}\right)=\mathrm{Dist}\left(S_{A},S_{B}\right)/\;\mathrm{Corr}\left(S_{A},S_{B}\right).$$

The $\mathrm{Dist}\left(S_{A},S_{B}\right)$ term corresponds to the geometrical distance from the points in the dotplot diagram of structure *S_A_* (reference) to the dotplot diagram of structure *S_B_* (alternative). The *Corr* term is related to the cross-correlation between histogram vectors built from the dotplot diagrams of both structures by adding the number of points in four different directions (X, Y, diagonal and anti-diagonal). Although the *Dist* term in DoPloCompare is somehow similar to AptaMat, it does not seem to be symmetrically defined, and hence it does not take into account the number of BPs in the alternative structure. On the other hand, the *Corr* term accounts for the similarity in the order and number of elements that both structures contain, even if the BPs involved in these elements are not the same in structures *S_A_* and *S_B_*.

### 2.4 Clustering

To fully challenge AptaMat, we evaluated its classification ability by applying a clustering approach on RNA structures belonging to different RFAM families (Berman *et al.*, 2000; Kalvari *et al.*, 2021). To avoid the potential bias induced by the secondary structure prediction method, we selected among the RFAM families those having more than 10 complete experimental 3D structures (see Supplementary Table S3 for a complete list of the retained RNAs), and we verified the absence of structural issues within their 3D structures. For families with more than 30 complete structures, we randomly chose 30 structures in order to have a balanced dataset. For the other families, we kept all the available structures. The resulting dataset consists in 14 RFAM families and 291 sequences. Finally, we computed the dot-bracket notation of each selected RNA secondary structure using ×3dna-dssr (Lu and Olson, 2003).

The clustering was performed using the affinity propagation method (Frey and Dueck, 2007). For a set $\left\{S_{1},\ldots ,S_{N}\right\}$ of *N* secondary structures, this method takes as input an affinity matrix $M_{\text{Affinity}}={\left(m_{ij}\right)}_{i,j=1}^{N}$, defined as

(5) $$m_{ij}=\text{exp}(-\frac{{\left(D_{\text{AM}}(S_{i},S_{j})\right)}^{2}}{2\sigma^{2}}),$$

built by computing the AptaMat distance for each pair of secondary structures. The scale parameter *σ* has been determined using Caliñski and Harabasz index (Caliñski and Harabasz, 1974) to optimize the clustering quality. Successively, an exchange of real-valued messages between data points is performed until a high-quality ensemble of representative examples and, thus, the corresponding clusters, gradually emerge. An advantage of this clustering method is that it does not need to fix the number of clusters, since an appropriate one is defined as a function of the submitted data.

## 3 Results and discussion

### 3.1 AptaMat as compared to currently used metrics

**Fig. 3.**
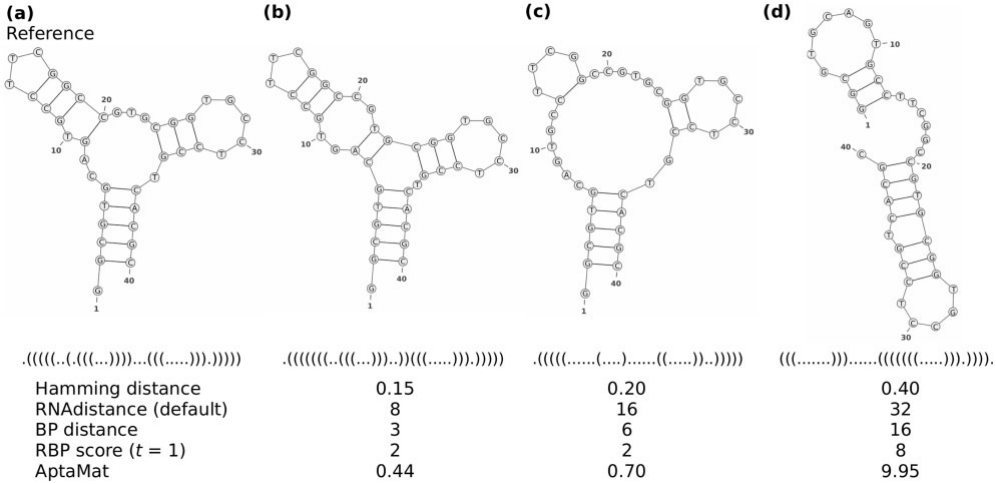
ssNA 7 shows the ability of AptaMat in comparing ssNAs secondary structures. The Hamming distance, the default RNAdistance, the BP distance and AptaMat indicate that the alternative structures (**b**), (**c**) and (**d**) are progressively farther from the reference secondary structure (**a**). For details about the results of the different RNAdistance options, we refer to Supplementary Figure S9 and Table S2

We used AptaMat to measure the distance between pairs of secondary structures for the ssNAs reported in Supplementary Table S1, and we compared the AptaMat distance with the Hamming distance, the BP distance and RNAdistance with the different available options. Among the selected structures, for ssNAs 2 and 7 (Figs 3 and 5) the Hamming distance, the default RNAdistance, BP distance and AptaMat distance between the alternative secondary structures and the reference one follow the same trend. In addition, the distance trend is also correctly predicted for ssNAs 4 and 5 (Supplementary Figs S6 and S7) by using either the Hamming distance, the default RNAdistance, or AptaMat distance. This shows the coherence between our method and the most used distance metrics when there is a clear difference between the compared secondary structures in terms of both dot-bracket notation and the trees used to calculate RNAdistance. However, concerning this latter metric, it is worth noticing that, depending on the chosen RNAdistance option, the results might differ in terms of the trend and the values obtained for some ssNAs (Fig. 2 and Supplementary Figs S4–S12).

We discuss here the results for ssNA 7 (Supplementary Table S1, Fig. 3 and Supplementary Fig. S9), for which we could gather three different alternative structures, allowing a more extensive analysis. The distances from the reference structure (a) progressively increase going from the alternative structure (b), obtained by RNAstructure (Reuter and Mathews, 2010) to (d), obtained by RNAfold (Gruber *et al.*, 2008b). Indeed, the reference secondary structure (a) made of a stem, a multi-branched loop, a bulge and two hairpin/stem loops is progressively lost. The alternative structure (b) is close to the reference: instead of the original G9-C20 BP, it has a BP between C7 and G17 and one between A8 and T18. This difference of 3 BPs leads to the transformation of the bulge in an internal loop and the reduction of the width of the multi-branched loop. Structure (c) has a much wider multi-branched loop because of the loss of 5 BPs, which also shortens the two hairpin/stem loops, with one of them becoming a bulge. Finally, structure (d) only conserves 2 hairpin/stem loops and the bulge but they do not involve the same positions as in the reference, for a total of 16 different BPs. In this case, all but the RNAdistance ‘W’ option (i.e. weighted coarse-grained structure translation followed by the string comparison) provided the same trend, although with different distance values.

However, sometimes the structural differences between two ssNAs are quite subtle and the Hamming distance, the BP distance and RNAdistance are not able to discriminate between structures. A striking example is represented by ssNA 1 (Supplementary Table S1 and Fig. 2), which has been taken from Ivry *et al.* (2009). This toy example is not based on the analysis of a proper ssNA sequence but it focuses directly on structures. As shown in Figure 2, the three structures compared to the reference differ from this latter and one from another. The three alternative structures have an additional bulge, which becomes progressively wider from structure (b) to structure (d) since the third BP progressively shifts towards the 5’ end. Nevertheless, the Hamming distance predicts the same distance to the reference for the three alternative structures since it counts the number of mismatches between the dot-bracket strings to compare regardless of the position of the nucleotides involved in BPs. As a result, any information about the structure is lost and different secondary structures with the same number of mismatching positions as compared to a reference structure will have the same Hamming distance from it. In ssNA 1 all the alternative structures have 2 mismatching positions, which, leading to a Hamming distance of 0.10 in all the cases. Similarly, the BP distance counts the number of different BPs between the two compared structures, which are 2 in all the comparisons of the alternative structures to the reference one. Even the inclusion of the relaxation term of the RBP score (*t* = 1, 5 or 10) does not allow the distinction between the different structures, giving a score of 1 for the three cases.

For what concerns RNAdistance, the structures of Figure 2 are considered to be equivalent, although with varying distances values, when using a full structure or a coarse-grained representation followed by either a string or a tree editing comparison (options ‘f’, ‘F, ‘c’ and ‘C’). Indeed, although RNAdistance takes into account the correlation between opening and closing positions of the dot-bracket strings, it might happen that the series of editing operations of two comparisons have an equivalent weight leading to the same RNAdistance, as it occurs in the example of Figure 2 (see Supplementary Fig. S1 for the details). Conversely, options ‘h’ and ‘H’, which are based on the HIT structure translation followed by rooted tree editing and string comparison, respectively, indicate that structures (b) and (d) are equally distant from structure (a), while structure (c) is farther (option ‘h’) or closer (option ‘H’) to structure (a) as compared to the other two. Option ‘W’ gives the opposite trend as the expected one and the only option providing the right trend is ‘w’, which consists in the weighted coarse-grained structure translation followed by the rooted tree editing comparison. Therefore, it appears clear that the choice of the appropriate RNAdistance option is non-trivial since it is strongly system dependent. On the opposite, both AptaMat and DoPloCompare are able to correctly and straightforwardly calculate the distance trend, with the first alternative structure being the closest to the reference and the third alternative structure being the farthest.

**Fig. 4.**
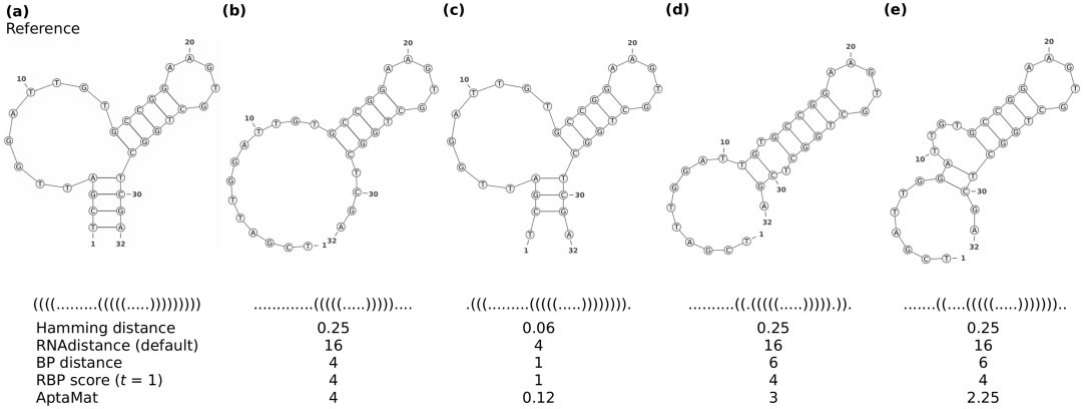
Reference (**a**) and alternative structures (**b**), (**e**) for ssNA 10. The Hamming distance, the default RNAdistance, BP distance, RBP score (with *t *=* *1) and AptaMat are computed using structure (a) as reference. For details about the results of the different RNAdistance options, we refer to Supplementary Figure S12 and Table S2

The more realistic examples corresponding to ssNAs 3, 6, 9 and 10 also show the limits of the currently used metrics as compared to AptaMat (Supplementary Figs S5, S8, S11, S12 and Figure 4). As mentioned before, the Hamming and BP distances will be the same if the alternative structures have the same number of mismatching positions and BPs, respectively, as compared to the reference one. However, depending on the number and the position of the mismatches, the structural difference might become highly relevant and lead to wrong conclusions about the similarity of a structure to a reference one. We discuss here ssNA 10, which highlights the issues arising from the Hamming distance, the BP distance and RNAdistance in a unique example. ssNA10 is a DNA aptamer, called pL1, binding to the Plasmodium vivax LDH, whose structure is described in the 5HRU PDB and consists in 4 base pairs followed by a bulge and a hairpin/stem loop (Fig. 4). Noteworthy, the bulge and, at minor extent, the hairpin/stem loop are implicated in the interaction with LDH. When the pL1 sequence is submitted to Mfold using a percent suboptimality of 100, in order to retrieve the maximum number of predicted structures, we obtained the alternative structures (b)–(e), with the former being the one corresponding to the one with the lowest ΔG (−3.50 kcal/mol) and the latter the one with the highest ΔG (−0.99 kcal/mol). When computing the distance of these alternative structures from the experimental one (reference structure (a)), all the considered metrics correctly indicate that structure (c) is the closest to the reference, since it only misses one base pair involving the 5′ and 3′ ends. The other alternative structures (e), (d) and (b) are progressively more distant from the experimental reference structure. Indeed, although it loses the four initial base pairs, structure (e), maintains the bulge, even if it is shorter than the one in the reference structure, and the hairpin/stem loop. Structure (d) misses the 4 base pairs, maintains the hairpin/stem loop, and it has a small internal loop instead of the bulge. Finally, structure (b) has only the hairpin/stem loop and it does not have the bulge or even an internal loop which would be needed for the interaction with the partner protein. This trend is correctly showed by AptaMat, while the Hamming distance and the default RNAdistance do not capture any differences from the reference structure for the alternative structures (b), (d) and (e). Only the RNAdistance options ’w’ and ’H’ do it in the expected way (Supplementary Fig. S12). The BP distance wrongly indicates that structures (d) and (e) have the same distance from the experimental structure, and, more importantly, structure (b) is suggested to be closer to the reference than structures (d) and (e). The inclusion of a relaxation parameter *t* (*t *=* *1, 5 or 10) within the RBP score does not lead to capture the expected differences (Fig. 4 and Supplementary Fig. S12).

AptaMat is also able to establish a more meaningful ranking of the alternative secondary structures in terms of distance from the reference as compared to the Hamming distance, the BP distance and the default RNAdistance in all the examples herein presented. This is important when investigating the effect of sequence mutations on the ssNAs secondary structure. In this context, ssNAs 3, 5, 6, 8 and 9 (Supplementary Table S1) show the limits of these methods as compared to AptaMat. Here we focus our discussion on ssNA 6, which has more alternative structures than ssNAs 3, 5 and 9, and more subtle modifications than ssNA 8. Thus, this example offers the possibility to deeply explore the differences between the considered metrics. ssNA 6 (PDB ID: 1NGO) has a simple hairpin/stem loop structure (Supplementary Fig. S8). The alternative structure (b) obtained by CentroidFold is correctly considered by the used metrics as the closest to the experimental structure (Hamming distance = 0.074, default RNAdistance = 2, BP distance = 2, and AptaMat = 0.091). AptaMat then indicates that the alternative structure (d) obtained by MC-Fold is closer to the reference (AptaMat distance = 0.20) than the alternative structure (c) obtained by RNAfold (AptaMat distance = 0.22), since the former only misses two pairs of bases (T5-G23 and T6-G22) while maintaining the overall structure. Conversely, structure (c) has two additional base pairs that lead to the loss of the characteristic loop of 1NGO (Supplementary Fig. S8). On the opposite, the Hamming distance fails in finding this difference, and the default RNAdistance suggests the opposite trend, with structures (c) and (d) having an RNAdistance of 6 and 8, respectively. It is worth noticing that none of the RNAdistance modes is able to correctly capture the expected trend (Supplementary Fig. S8). In this context, the BP distance also fails in doing so: although indicating that structure (c) is the farthest from structure (a), it cannot distinguish between structures (b) and (d). The use of the relaxed RBP score does not change the situation here.

Similar conclusions are applicable to ssNA 3 and 8 (Supplementary Figs S5 and S10), though for this latter the BP distance succeeds in finding the right trend. For ssNAs 5 and 9 (Supplementary Figs S7 and S11) the Hamming and the BP distances indicate an opposite and inadequate ranking of the two alternative structures because of the different number of mismatches.

The overall better performance of AptaMat in ranking the alternative secondary structures in terms of distance from a reference, as compared to the Hamming distance, the BP distance or RBP score, and RNAdistance, is particularly evident for structures that are close to the reference one, which turn out to be more difficult to properly rank. The ability of AptaMat in doing so is due to the higher weight given by our algorithm to the relative position of the base pairs. This leads to focus on the global secondary structure more than on the local differences from the reference secondary structure. As previously mentioned, this is of particular importance for the comparison of ssNAs, since their function highly depends on their global 3D structure and only to a minor extent on local sequence information.

In addition, together with the better performance shown here as compared to the other discussed metrics, AptaMat has the advantage of being easily applicable since there is no need to choose any parameter as for RNAdistance or the RBP score. Moreover, it can be used for the comparison of sequences with different lengths, which is not possible for the Hamming distance and it is not recommended for the BP or RBP distances. Finally, thanks to the use of dotplots to describe the structural information, and contrary to RNAdistance, AptaMat allows the handling of pseudo-knots.

**Fig. 5.**
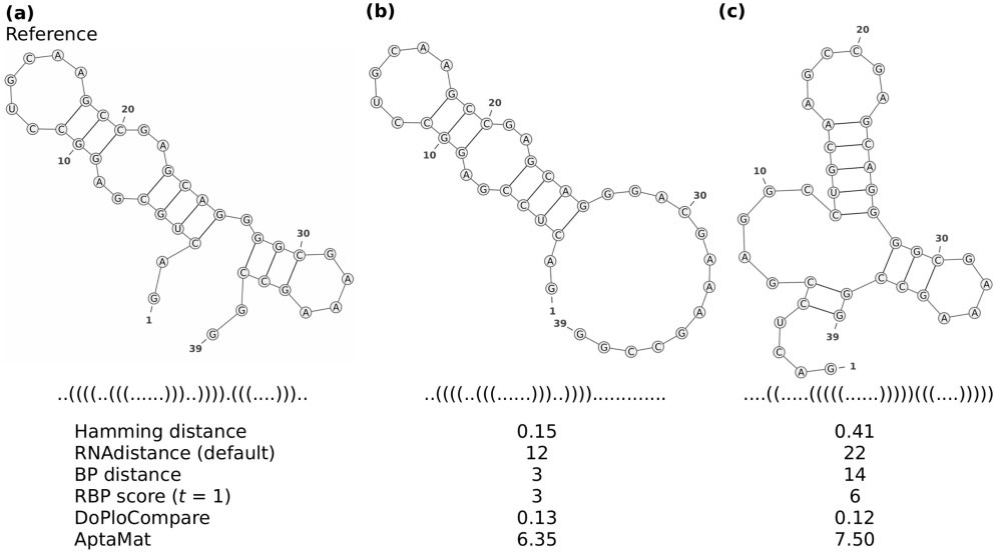
Reference (**a**) and alternative structures (**b**) and (**c**) for ssNA 2. The Hamming distance, the default RNAdistance, BP distance, RBP score (with *t *=* *1) and AptaMat are computed using structure (a) as reference. For details about the results of the different RNAdistance options, we refer to Supplementary Figure S4 and Table S2

The analysis of the alternative structures ranking relative to the reference structure highlight the limits of DoPloCompare as compared to AptaMat. SsNAs 2, 4 and 5 (Fig. 5, Supplementary Figs S6 and S7) have a DoPloCompare trend opposite not only to AptaMat but also to the Hamming distance and RNAdistance. We argue that this is due to the *Corr* term in DoPloCompare, which, as we mentioned before, accounts for the similarities in the number and order of the elements (stems, loops, etc.) in the compared structures. In the three previous examples, the structures that are found to be closer to the reference one are those having a more similar number of elements, despite the fact that the base pairs involved in these elements are not the same. For example, if we consider ssNA 2 (Fig. 5), we can clearly see that the alternative structures (b) and (c) are both structurally far from the reference structure (a). However, the structure (b) is closer to the reference (a) (Hamming distance = 0.15, default RNAdistance = 24, BP distance = 3 and AptaMat = 6.35) than the alternative structure (c) (Hamming distance = 0.41, default RNAdistance = 26, BP distance = 14 and AptaMat = 7.50), as correctly indicated by the Hamming distance, the default RNAdistance, the BP distance and AptaMat. Indeed, structure (b) maintains the secondary structure of the reference except for 3 missing base pairs (G28-C37, G29-C36 and C30-G35), while structure (c) has four additional base pairs (C5-G39, C6-G38, C12-G27, U13-G26), leading to a significant change in the global structure. DoPloCompare indicates that this latter structure is closer to the reference (DoPloCompare = 0.12) than structure (b) (DoPloCompare = 0.13), because structure (c) has two hairpin/stem loops and an internal loop as structure (a), while structure (b) only has a hairpin/stem loop and an internal loop. However, the global structure (c) differs from those in structure (a), because of a different base pairs pattern. In addition, the DoPloCompare scores are close to 0, suggesting a high similarity of the alternative structures to the reference one, which is clearly not the case as indicated by the other metrics. Similar observations can be done for ssNAs 4 and 5 (Supplementary Figs S6 and S7). Furthermore, looking at the DoPloCompare scores obtained for ssNAs 1–5, it seems that they depend on the sequence length: although the alternative structures of ssNAs 1 (Fig. 2) are globally close to the reference one, they show a DoPloCompare score which is higher than those obtained for ssNAs 2–5, where the alternative structures are very far from the reference, as also showed by the default RNAdistance and AptaMat.

### 3.2 Considering structural ensembles within AptaMat

Because of the high flexibility of ssNAs, it is not unusual to have an ensemble of possible foldings (Ganser *et al.*, 2019; Herschlag *et al.*, 2015) for a given ssNA sequence, each having a different associated weight. This can change as a function of the experimental conditions (ions concentration, pH, temperature, …) or in the presence of a molecule recognized by the ssNA (ligand) (Haller *et al.*, 2011). Therefore, it might be interesting not only to independently compare each alternative structure to a reference one but also to consider simultaneously the whole conformational ensemble and its associated distribution when performing the comparison to a reference structure. This can be done within AptaMat by specifying the option ‘ – *ensemble*’, which allows the computation of the AptaMat distance according to Equation 2, after having provided the alternative structures and the associated weights. The reference structure might be a consensus structure or it can be the experimental structure to which predicted co- or suboptimal structures can be compared to evaluate the reliability of the prediction, as for ssNA 10. Alternatively, the reference structure can be the most probable ssNA structure obtained by changing the experimental conditions, which will allow the study of the effect of the resulting conformational changes by taking into account the whole structural ensemble. For example, experimental data on the transactivation response (TAR) RNA of the HIV-1 transactivator protein TAT showed that it can adopt different similar conformations in the absence of a ligand (PDB code 1ANR, Table 1). Conversely, when TAR binds a peptide mimicking the TAT protein (PDB code 2KDQ), only one conformation is sampled. We applied both the default and the weighted AptaMat algorithm, using the peptide-bound structure as a reference and we used as weights the relative frequencies of each conformation. Table 1 reports the obtained results. The weighted AptaMat distance of the ligand-free conformational ensemble from the peptide-bound structure is 0.16, indicating minor conformational changes. Moreover, looking at the individual AptaMat distances, we can observe that, among the alternative structures, the third one (weight = 0.15) is identical to the peptide-bound structure. Thus, it seems that the presence of the ligand stabilizes a minor conformation of the ligand-free structural ensemble. In addition, the most probable ligand-free conformation is the closest to the reference one, suggesting that only a small conformation change occurs.

**Table 1.** Comparison of the ligand-free structural ensemble of the TAR RNA to the peptide-bound TAR RNA structure

No.	Structure	Counts^a^	Weight	AptaMat
1	(((((….((((……)))).)))))	9	0.45	0.10
2	.((((….((((……)))).)))).	5	0.25	0.22
3	((((((…((((……))))))))))	3	0.15	0.00
4	.((((…..(((……)))..)))).	1	0.05	0.35
5	((((……((((…).)))…))))	1	0.05	0.61
6	.(((((…((((……))))))))).	1	0.05	0.10
	Weighted AptaMat			0.16

*Note*: ((((((…((((……)))))))))), from PDB code 2KDQ.

a Out of 20, from PDB code 1ANR.

### 3.3 Clustering RNAs from RFAM families

In order to further challenge AptaMat, we tested its ability in clustering sequences belonging to different RFAM families. We considered only RFAM families with experimental 3D structures to avoid a bias related to the secondary structure prediction, even if we might end up with incomplete structures, because of missing residues at the extremities, generating subclusters. For families having a high number of experimental structures available, such as tRNA and the bacterial small subunit ribosomal RNA, we randomly selected 30 structures. In addition, we discarded the families with less than 10 available structures. The final dataset consisted in 291 structures and 14 RFAM families (Supplementary Table S3). We used the affinity propagation method (Frey and Dueck, 2007) to perform the clustering (see Section 2 for further details), since it does not need to preliminary fix the number of clusters. To quantitatively evaluate the results we computed the silhouette score, which indicates the separation between clusters and which assumes values between −1 and 1, with higher values coming from well-distinguishable clusters and lower values from difficult-to-separate clusters. We also computed the clustering accuracy, calculated as the sum of the diagonal elements of the confusion matrix divided by the total number of sequences, and the adjusted random score, which allows the determination of the similarity between clusters. The obtained silhouette, adjusted random scores and accuracy are of 0.55, 0.84 and 0.82, respectively, indicating the good quality of the clustering.

**Fig. 6.**
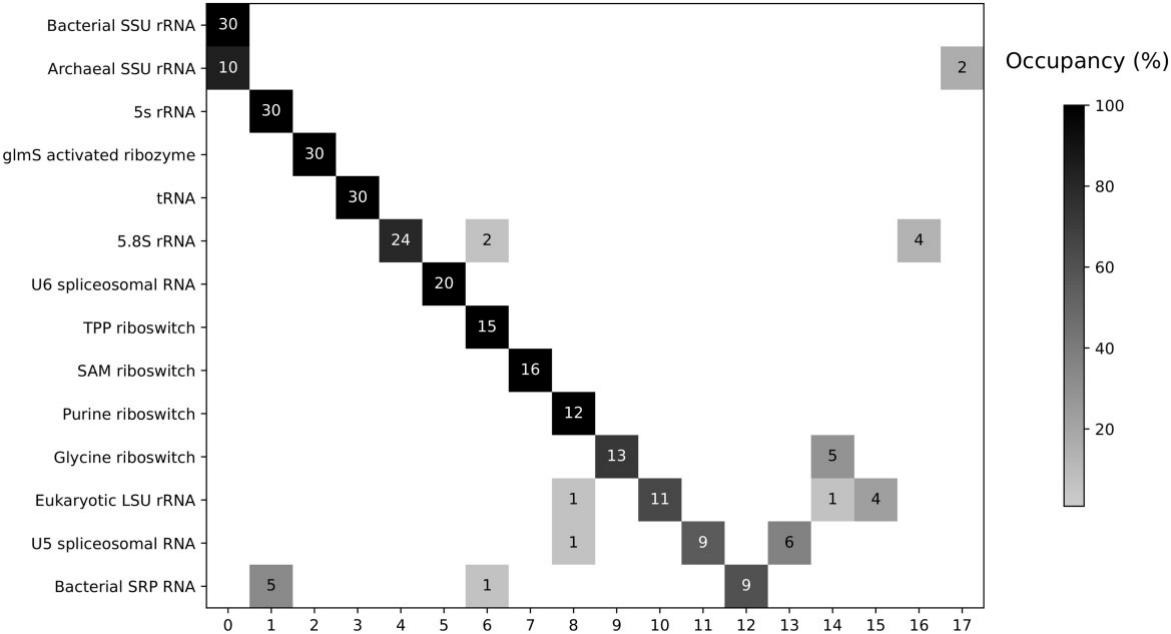
Graphical representation of the clustering performed on selected structures belonging to 14 RFAM families by using AptaMat as distance metric and the affinity propagation as clustering algorithm. The number of structures contained in each cluster is indicated in the cluster square. The color gradient corresponds to the occupancy of each cluster

Figure 6 shows the results of the clustering as a confusion matrix. As it can be seen, the clustering using AptaMat distance is globally able to recover the expected subdivision within the different RFAM families. For 8 out of the 14 RFAM families, namely the bacterial small subunit (SSU) rRNA, 5S rRNA, tRNA, glmS activated ribozyme, U6 spliceosomal RNA, TPP riboswitch, purine riboswitch and SAM riboswitch a unique cluster is identified. In addition 13 out of 17 clusters are independent, i.e. they are not shared between different families. It should be noticed that the structures for the bacterial SSU rRNA are not complete (about 1000 versus the expected 1500 nucleotides) and the related cluster 0 includes not only the bacterial SSU rRNA but also most of the archaeal SSU rRNA structures, since in both cases this ribosomal subunit corresponds to the 16S rRNA. The only two archaeal SSU rRNA structures not included in cluster 0, but isolated in cluster 17, correspond to the whole 16S rRNA. For the other families, we can clearly distinguish a major cluster and one or more minor clusters, whose presence can be easily explained. More in detail, for the 5.8S rRNA family, most of the structures belong to the unique cluster 4. The two structures belonging to cluster 6 (2WWB chain D and 2WWA chain D) are significantly shorter (about 60 nucleotides), probably because of the experimental structural resolution, making them closer to the cluster grouping the TPP riboswitch. Cluster 16 contains 3 5.8S rRNA from *Drosophila melanogaster*, which has a shorter 5.8S rRNA (123 nucleotides) as compared to other species and a human 5.8S rRNA with multiple missing residues at both the 3′ and 5′ ends, leading to a significantly shorter sequence. The glycine riboswitch family is split into two clusters (9 and 14), with cluster 9 containing only the synthetic domain II of the riboswitch, while cluster 14 contains a more complete structure from different species. The U5 spliceosomal RNA family is split in 3 clusters: two major unique clusters (11 and 13), whose separation depends on the presence of multiple missing nucleotides at both the 5′ and 3′ extremities for the structures of cluster 11, and a minor one (cluster 8), which corresponds to the purine riboswitch family cluster. In this cluster, we found the U5 spliceosomal RNA from *Saccharomyces cerevisiae* which has an unusual insertion in its sequence (Mitrovich and Guthrie, 2007). As expected, we observed that the eukaryotic large subunit (LSU) rRNA family available structures do not describe the whole LSU rRNA, but either part of the 25S or 26S rRNA (cluster 10), or part of 28S rRNA (cluster 15). In addition, clusters 8 and 14 contain two LSU rRNA chains of a highly atypical *Euglena gracilis* rRNA (Matzov *et al.*, 2020) (6ZJ3 chains LB and LC, respectively). Finally, the bacterial small recognition particle RNA (SRP RNA) is divided into cluster 12 containing only 4.5S RNA, cluster 1 containing 7S.S RNA, which cannot be distinguished from the 5S RNA, and cluster 6, corresponding to the TPP riboswitch, which also contains a 7S.S RNA from *Methanocaldococcus jannaschii*.

## 4 Conclusion

Being able to compare ssNAs secondary structures is fundamental to understand the function and evolution of this kind of biomolecules, to design ssNAs with a desired secondary structure or even to predict the conformational effects of sequence mutations. To that extent, in this work we present AptaMat, a new matrix-based algorithm capable of comparing pairs of aligned ssNAs secondary structures, with *L* being the alignment length. The alignment can be performed both externally or internally (with RNAlign2D). AptaMat then takes as input the two aligned ssNAs structures in the dot-bracket notation and, for each of them, creates a matrix of size *L *×* L*, named $A=(a_{ij})$ and $B=(b_{ij})$. The (*i*, *j*)th entry of the matrix is either equal to 1 if the nucleotide in position *i* is paired with the nucleotide in position *j* or 0 if the nucleotides in positions *i* and *j* are not paired or in presence of a gap. Then, for each 1≤i<j≤L such that *a_ij_* = 1, the Manhattan distance to the closest entry equal to 1 in matrix *B*, and vice versa, is calculated. The distances between all the closest pairs are summed up and a cost of 1 is associated to each gap. Finally, the normalization by the total number of cells containing 1 in both matrices is performed, leading to AptaMat distance.

We compared AptaMat to some of the most used metrics for ssNAs secondary structures comparison, namely the Hamming distance, the BP distance, the relaxed RBP score, the different options of RNAdistance and a more recent approach based on image processing, DoPloCompare, by Ivry *et al.* (2009). In order to do this, we chose five structures taken from the examples reported in the work by Ivry *et al.* (2009) and five structures taken from the PDB database.

We showed that AptaMat is able to properly distinguish between different structures, presenting a higher sensitivity and a more adequate ssNAs structures ranking ability as compared to the other considered metrics. Moreover, it is easy to interpret, it can deal with sequences of different lengths, or the presence of pseudoknots, and it is less affected by the ssNA length than the other considered metrics. Additionally, AptaMat is easy to implement and to manipulate. Indeed, we plan to extend its usage to peculiar structures, such as G-quadruplex, which represent a challenging task in nucleic acids modeling. Finally, we used the AptaMat distance as a metric within a clustering study of 291 structures belonging to 14 RFAM families, and we showed its ability to correctly recover the different RFAM families and to detect species peculiarities. These results suggest that AptaMat could also be potentially used for ssNAs sequences annotation, one of the most relevant and challenging bioinformatics tasks.

## Supplementary Material

btac752_Supplementary_Data

## Data Availability

The python code for AptaMat is available at https://github.com/GEC-git/AptaMat.git.
