# Supplementary material for: AptaMat: a matrix-based algorithm to compare single-stranded oligonucleotides secondary structures
Source: Bioinformatics. 2022 Nov 28;39(1):btac752. doi: 10.1093/bioinformatics/btac752 (PMC9805580; doi:10.1093/bioinformatics/btac752)
Supplement: btac752_Supplementary_Data [file btac752_supplementary_data.pdf]

Supplementary Material for ‘AptaMat: a matrix-based algorithm to compare single stranded oligonucleotides secondary structures’

Thomas Binet[1], Bérangère Avalle[1], Miraine Dávila Felipe[2,*] and Irene Maffucci[1,*]
[1]Université de technologie de Compiègne, UPJV, CNRS, Enzyme and Cell Engineering, Centre de recherche Royallieu - CS 60 319 - 60 203 Compiègne Cedex
[2]Université de technologie de Compiègne, LMAC (Laboratory of Applied Mathematics of Compiègne), CS 60 319 - 60 203 Compiègne Cedex
*To whom correspondence should be addressed.

Contents

List of Tables

Table S1 ssNAs test set nomenclature S2
Table S2 RNAdistance set S2
Table S3 Selected RFAM families and structures for the clustering study S3

List of Figures

Figure S1 Vienna Tree Representation S8
Figure S2 AptaMat flowchart S9
Figure S3 AptaMat algorithm S10
Figure S4 Results for ssNA 2 . . . . . S11
Figure S5 Results for ssNA 3 . . . . . S11
Figure S6 Results for ssNA 4 . . . . . S12
Figure S7 Results for ssNA 5 . . . . . S13
Figure S8 Results for ssNA 6 . . . . . S14
Figure S9 Results for ssNA 7 . . . . . S14
Figure S10 Results for ssNA 8 . . . . . S15
Figure S11 Results for ssNA 9 . . . . . S16
Figure S12 Results for ssNA 10 . . . . . S16

Table S1: ssNAs test set nomenclature.

No.	Code PDB[a]	Length	Reference dot-bracket notation
ssNAs from [Ivry *et al.*, 2009]			
1	NA	20	.(((............))).
2	NA	39	..((((..(((......)))..)))).(((....)))..
3	NA	43	.((((((((((((...((.......))...))))))))).)))
4	NA	56	....((((.(((((.......))))).))))....((((........)))).))))
5	NA	127	((((((((...(((((((((((((((..((((((.((((((((.(((.((....((((..((((... ...))))..))))))))))))))))).)).)))).)))))).))))))))).))))))))
ssNAs from PDB database			
6	1NGO	26	(((((((((((.....)))))))))))
7	3HXO	40	.(((((..(.(((...))))...(((.....))).)))))
8	1SNJ	36	(((((((((((..))))((((..))))..)))))))
9	2VJU	35	((..((((((((....)))).))))......))..
10	5HRU	32	((((.........(((((.....)))))))))

[a]The PDB code is not available for the structures taken from [Ivry *et al.*, 2009]

Table S2: RNAdistance test set calculation.

ssNAs No.	Structure	Distance calculation method							
		**f**	**h**	**w**	**c**	**F**	**H**	**W**	**C**
1	b	4	8	10	3	2	24	46	3
	c	4	14	22	3	2	23	40	3
	d	4	8	30	3	2	24	42	3
2	b	12	18	21	3	6	36	57	3
	c	22	26	25	6	13	18	20	4
3	b	24	32	38	6	19	46	69	6
	c	26	38	37	1	21	46	55	1
4	b	26	32	51	10	16	24	36	6
	c	44	44	47	9	26	36	43	8
5	b	60	74	80	14	38	58	74	12.5
	c	62	66	79	14	39	67	133.5	12
6	b	2	6	6	3	2	24	15	3
	c	6	10	11	3	4	24	18	3
	d	4	24	18	3	4	24	18	3
7	b	8	8	18	1	6	8	18	1
	c	16	18	24	3	8	18	78	3
	d	32	32	40	9	15	24	35	7
8	b	8	8	10	0	4	8	10	0
	c	24	24	37	5	12	51	91	5
	d	28	28	45	5	14	46	99	5
9	b	8	12	29	3	6	12	38	3
	c	8	12	30	3	4	13	57	3
10	b	16	16	39	3	8	30	93	3
	c	4	4	3	0	2	4	3	0
	d	16	16	29	1	8	15	29	1
	e	16	18	21	0	8	16	21	0

Table S3: RFAM families and structures for the clustering study.

FAMILY	RFAM	PDB	CHAIN
glmS glucosamine-6-phosphate activated ribozyme	RF0234	4MEG	B
		3G8T	P
		3L3C	R
		2HO7	B
		3G9C	R
		3G9C	Q
		3G8T	Q
		2HO6	B
		2H0W	B
		3G9C	P
		3L3C	Q
		3G8T	S
		2GCS	B
		3L3C	P
		2NZ4	S
		3G96	R
		2NZ4	Q
		3B4C	B
		3B4B	B
		3G96	P
		2Z74	B
		2GCV	B
		2NZ4	P
		2H0Z	B
		3G9C	S
		3G8S	R
		3G8S	Q
		2NZ4	R
		3G8T	R
		2H0S	B
SAM riboswitch	RF00162	5FK3	A
		5FK1	A
		4B5R	A
		5FKG	A
		2YGH	A
		5FK6	A
		5FKH	A
		5FKF	A
		5FKE	A
		3IQP	A
		5FK4	A
		2YDH	A
		5FK5	A
		4AOB	A
		5FK2	A
		5FKD	A
U6 spliceosomal RNA	RF00026	6J6N	E
		6J6G	E
		6FF7	6
		6BK8	6
		6ICZ	F
		5WSG	E
		6ID0	F
		6ID1	F
		5GM6	E
		7W59	F
		7DCO	F
		5LQW	6
		6QDV	6
		3JB9	N
		7W5A	F
		6ZYM	6

		7W5B	F
		6FF4	6
		5GMK	E
		6J6Q	E
U5 spliceosomal RNA	RF00020	6ID0	B
		6AH0	B
		7B9V	5
		6FF4	5
		6ICZ	B
		6AHD	B
		6FF7	5
		5O9Z	5
		7ABF	5
		7AAV	5
		6ID1	B
		5MQF	5
		7ABG	5
		3JB9	C
		6ZYM	5
		7ABI	5
Purine riboswitch	RF00167	6UC7	B
		2XNZ	A
		3IVN	B
		2XO1	A
		3IVN	A
		3LA5	A
		2XO0	A
		5SWD	B
		5SWD	A
		2XNW	A
		5E54	B
		5E54	A
TPP riboswitch	RF00059	3K0J	E
		3D2G	A
		7TZS	X
		3D2X	B
		2HOO	A
		3K0J	F
		2GDI	Y
		3D2G	B
		2CKY	B
		2CKY	A
		7TZR	Y
		7TZR	X
		7TZS	Y
		3D2X	A
		2GDI	X
Eukaryotic large subunit ribosomal RNA	RF02543	7R81	A1
		4V8P	D1
		4V8P	A1
		5JCS	x
		6XU6	A5
		4V6W	A5
		4V7H	B5
		6XU8	A5
		5FL8	x
		4V8P	H1
		7OLC	1
		4V8P	F1
		6ZJ3	LC
		7OLD	1
		6XU7	A5
		4V7E	Aa
		6ZJ3	LB

5s rRNA	RF00001	6SPB	B
		6CFK	1B
		4V4A	B9
		6T83	Bb
		4V4X	BB
		4V5K	BB
		4V53	BA
		4WWW	RB
		4U4U	3
		3JAI	7
		4V4W	B9
		1VY5	DB
		3JCE	B
		7S0S	i
		6SPD	B
		3CCE	9
		4V66	BA
		4V9C	DB
		7Z20	a
		1YIJ	9
		6ZTO	BB
		6DZP	B
		4D5Y	4
		3JAN	7
		6OLZ	A4
		4U3M	7
		5E81	1J
		6YHS	5
		7RQC	1B
		7F5S	L7
5.8S ribosomal RNA	RF00002	4U3U	8
		6SXO	L8
		6T83	3b
		4V8P	C2
		6IP6	1C
		4U53	8
		2WWB	D
		7TOO	A58S
		2WWA	D
		6GQV	4
		6XU7	A8
		5ON6	AT
		6FTJ	w
		6GZ4	A3
		4V7H	B4
		4V6W	A8
		7OLC	4
		6I7O	YS
		4U55	8
		5TBW	4
		6N8J	3
		6Y2L	L8
		6XU6	A8
		6Z6K	C3
		3J7P	8
		4U4U	4
		6FTI	w
		5DGE	4
		4V8M	BC
		4U6F	4
		5GAK	B
		3A2K	D
		4V4Z	AD
		4V7J	Bv

tRNA		4WSD<br>6ZJ3<br>6WDD<br>4V7L<br>5LMV<br>2RD2<br>7SS9<br>3WFR<br>3L0U<br>4V4S<br>7SYR<br>5T2C<br>4WT1<br>7PAM<br>6WD4<br>6RFL<br>4V9R<br>7NSQ<br>4V8B<br>4WQR<br>6N1D<br>7ST2<br>5V6X<br>6WD9<br>7PIT<br>6Z6N	1K<br>S4<br>6<br>AX<br>Z<br>B<br>6<br>A<br>A<br>AV<br>i<br>An<br>3L<br>6<br>6<br>U<br>AX<br>8<br>CD<br>1K<br>BATN<br>5<br>C<br>6<br>8<br>CC
bacterial small subunit ribosomal RNA	RF00177	6OGI<br>4KVB<br>4X66<br>6GZZ<br>4U24<br>5J8A<br>4KHP<br>2UUC<br>5MY1<br>4V5Q<br>5LZD<br>6CAQ<br>7BOH<br>7DUG<br>4LF6<br>7ABZ<br>7OIG<br>6ENU<br>4V5J<br>4V6K<br>6YEF<br>5MDY<br>5UYP<br>7M4X<br>4V9C<br>4DR7<br>5IWA<br>1HNW<br>4V61<br>5J5B	3<br>A<br>A<br>A4<br>AA<br>AA<br>A<br>A<br>A<br>AA<br>a<br>A<br>A<br>A<br>A<br>2<br>2<br>a<br>AA<br>BA<br>a<br>2<br>A<br>a<br>AA<br>A<br>A<br>A<br>AA<br>AA
Bacterial small signal recognition particle RNA	RF00169	5AKA<br>5NCO<br>1LNG<br>2XXA<br>4XCO<br>2V3C<br>4XCO<br>5GAG	7<br>1<br>B<br>F<br>M<br>M<br>E<br>1

		5GAF	1
		2V3C	N
		5GAH	1
		2XKV	B
		1Z43	A
		2XXA	G
		3ZN8	G
Archaeal small subunit ribosomal RNA	RF01959	7ZAI	2
		4V6U	A2
		4V4N	B2
		6SWD	2
		6TMF	A
		5JBH	2
		7ZAH	2
		7ZHG	2
		5JB3	2
		6SW9	2
		6SWC	2
		7ZAG	2
Glycine riboswitch	RF00504	3OXE	A
		3OWZ	A
		3OX0	A
		3OXD	A
		3OWI	B
		3OXJ	A
		3OXE	B
		3P49	A
		3OXM	A
		3OXM	B
		3OWI	A
		3OXB	A
		3OX0	B
		3OWW	A
		6WLM	A
		6WLT	A
		6WLL	A
		6WLL	U

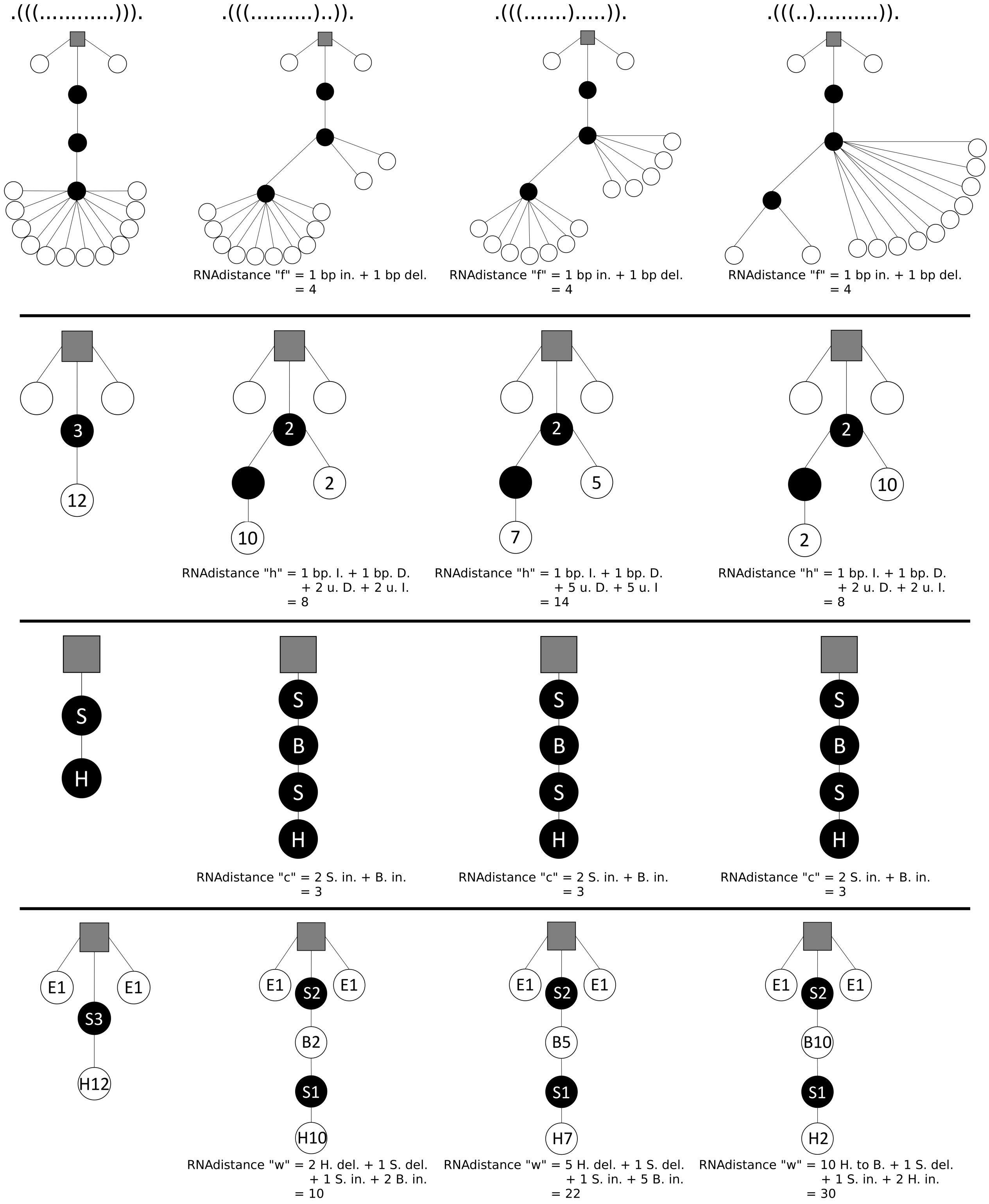

Figure S1: Dot-bracket and tree representations of ssNA 1 (**a**), and the alternative structures (**b**), (**c**), and (**d**). The computation of RNAdistance in full tree edit ("f"), HIT tree edit ("h"), coarse grain tree edit ("c") and weighted coarse grain tree edit ("w") for each of the alternative structures is provided under the corresponding trees. When performing full tree edit ("f") and HIT tree edit ("h") operations, "bp" means base pair while "u" means unpaired. While performing the coarse grain tree edit ("c") and weighted coarse grain tree edit ("w") operations, the modification matrix is wider as these representations consider structural motifs :"H" for helix, "B" for bulge,"I" for interior loops, "M" for multiloops, "S" for stacks and "E" for external bases. The edit operation costs may vary according to the modifications and the cost matrix used. The edit operations are indicated as "in." for an insertion, "del." for a deletion and "to" for a mutation.

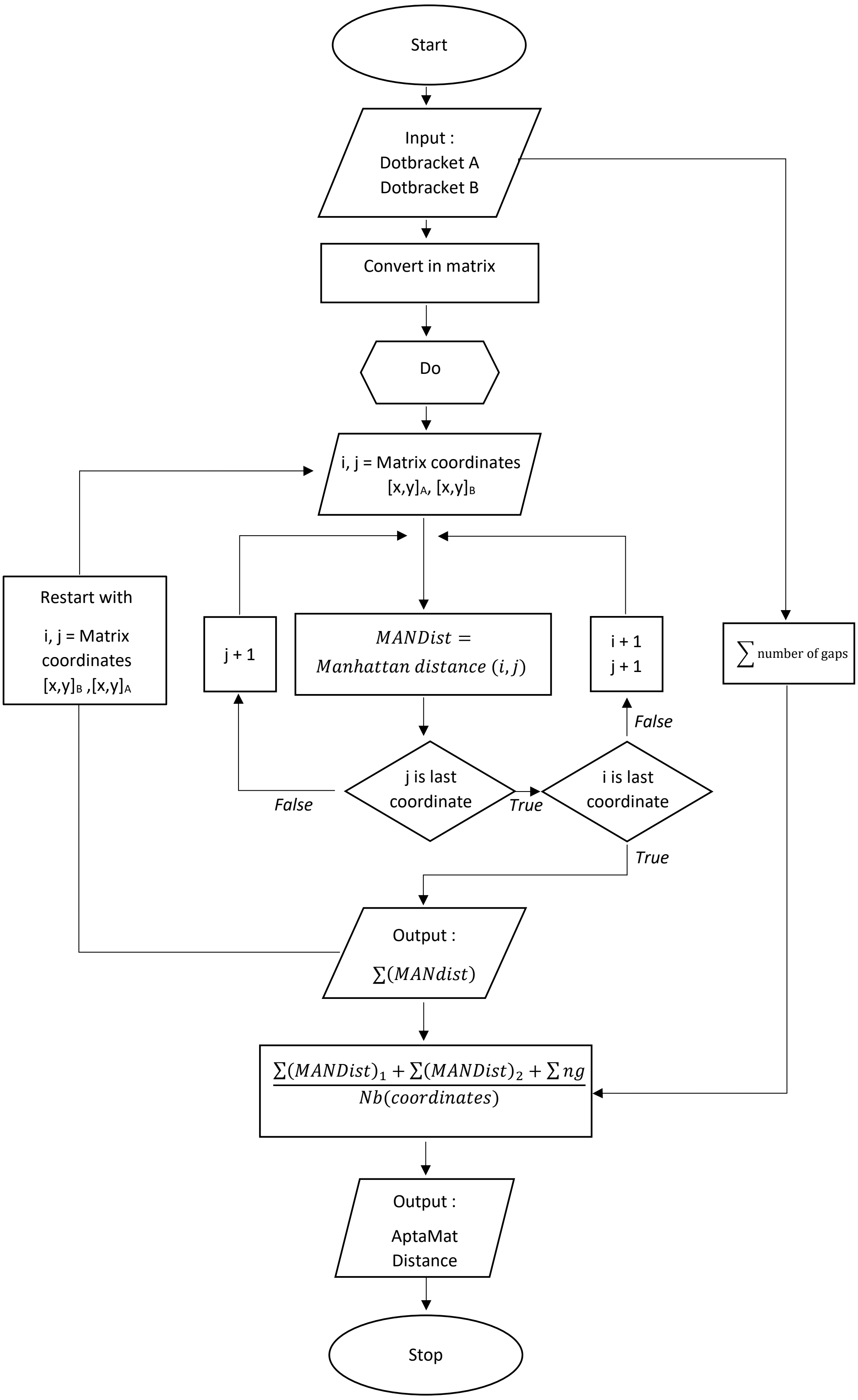

Figure S2: Flowchart of AptaMat algorithm

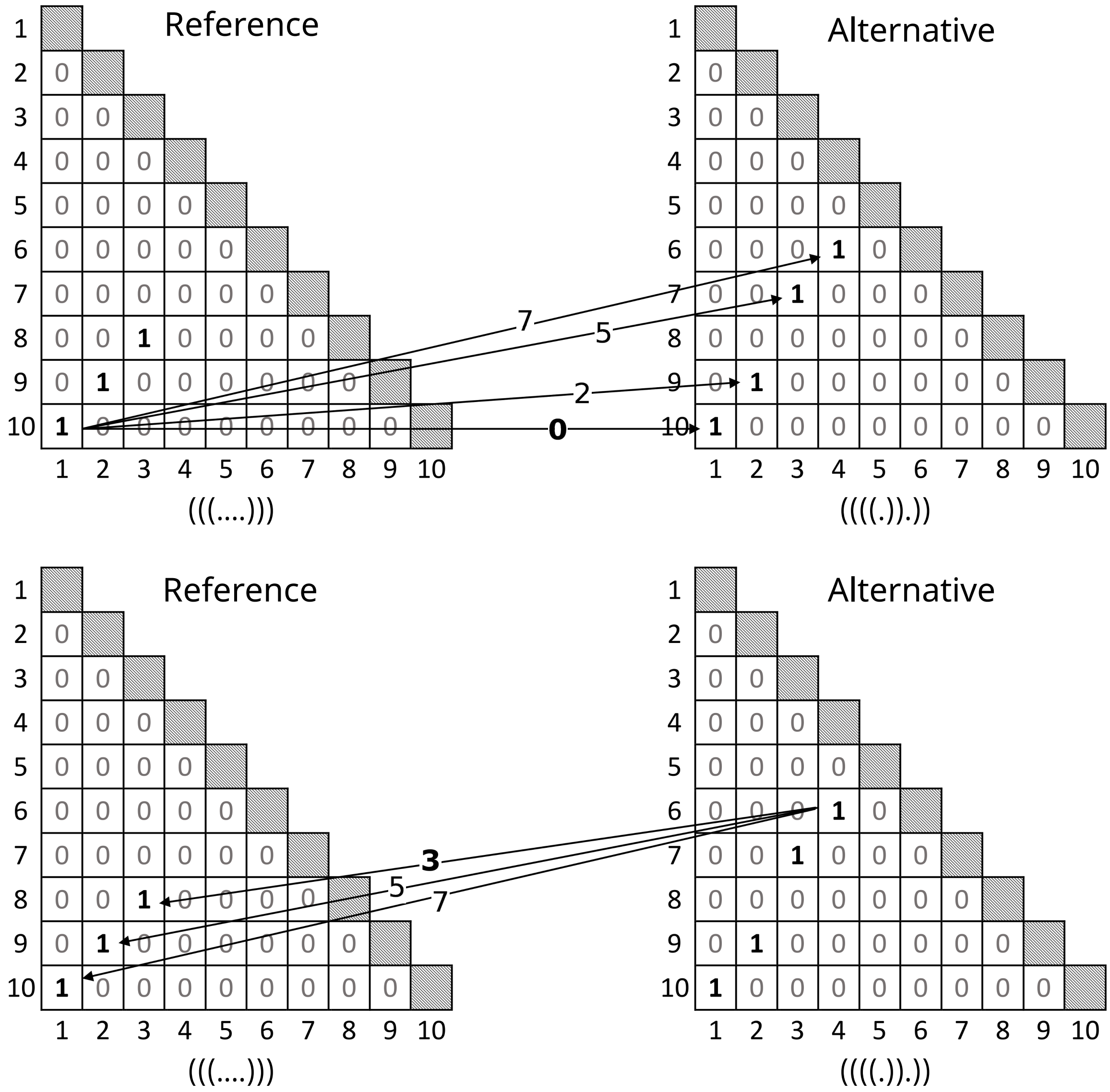

Reference

Alternative

Coordinate	1-10	2-9	3-8	Lowest
**1-10**	0	2	4	0
**2-9**	2	0	2	0
**3-7**	5	3	1	1
**4-6**	7	5	3	3
**Lowest**	0	0	1	

$$\frac{\sum Lowest\ ManDist\ (Ref,\ Alt) + \sum LowestManDist(Ref, Alt)\ + \sum ng}{nb\ coordinates}$$

$$= \frac{(\ 0 + 0 + 1\ ) + (0 + 0 + 1 + 3) + 0}{7} = \ 0.714$$

Figure S3: Detailed AptaMat calculation for a simple example. a) Matrix representation of 2 secondary structures and the performed comparison shown by the arrows and their associated Manhattan distances. For each "1" from a matrix we find the nearest "1" from the second matrix in Manhattan distance, in both directions Reference –> Alternative, then Alternative –> Reference. b) The Manhattan distances between every pair of "1"s. c) The general formula to compute AptaMat and the resulting value for this example.

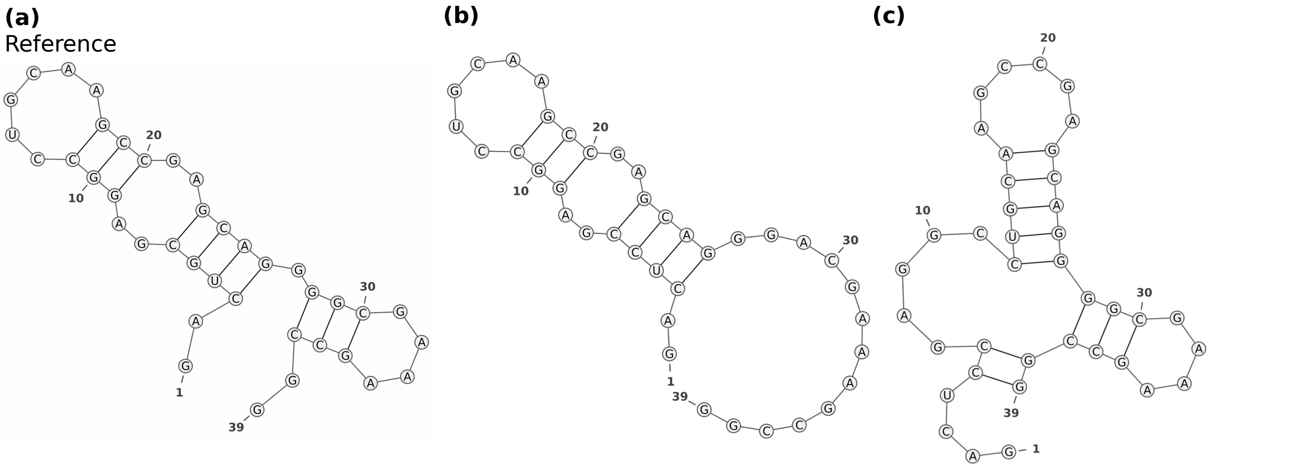

	(a) Reference	(b)	(c)
	..((((..(((......)))..)))).(((....)))..	..((((..(((......)))..))))).............	....((.....(((((......)))))(((....)))))
Hamming distance		0.15	0.41
RNAdistance "f" (default)		12	22
"h"		18	26
"w"		21	25
"c"		3	6
"F"		6	13
"H"		36	18
"W"		57	20
"C"		3	4
BP distance		3	14
RBP score (t = 1)		3	6
(t = 5)		3	2
(t = 10)		2	2
DoPloCompare		0.13	0.12
AptaMat		6.35	7.50

Figure S4: Graphical representations, dot-bracket notations of the representative structure of ssNA 2 (**a**) and the alternative structures (**b**) and (**c**). The Hamming distance, RNAdistance, BP distance, RBP score using t value within 1, 5 and 10, DoPloCompare, and AptaMat distances from the reference are also reported.

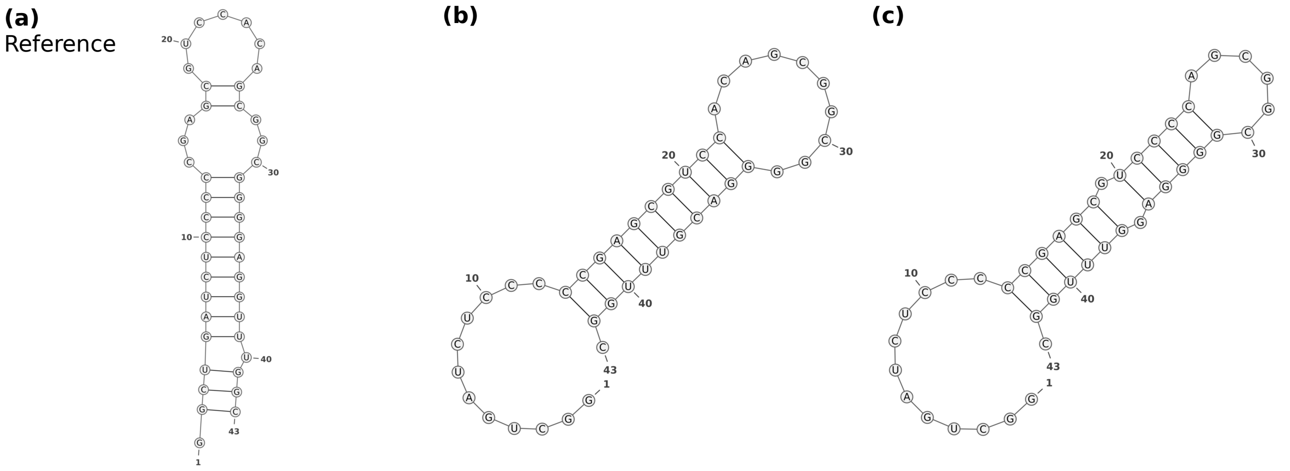

	(a) Reference	(b)	(c)
	.(((((((((((...((.......))...))))))))).)))	...........((((((((((..........)))))))))).	...........((((((.(((((......)))))).))))).
Hamming distance		0.56	0.56
RNAdistance "f" (default)		24	26
"h"		32	38
"w"		38	37
"c"		6	1
"F"		19	21
"H"		46	46
"W"		69	55
"C"		6	1
BP distance		24	25
RBP score (t = 1)		7	7
(t = 5)		2	2
(t = 10)		1	1
DoPloCompare		0.36	0.19
AptaMat		10.8	10.5

Figure S5: Graphical representations, dot-bracket notations of the representative structure of ssNA 3 (**a**), and the alternative structures (**b**) and (**c**). The Hamming distance, RNAdistance, BP distance, RBP score using t value within 1, 5 and 10, DoPloCompare, and AptaMat distances from the reference are also reported.

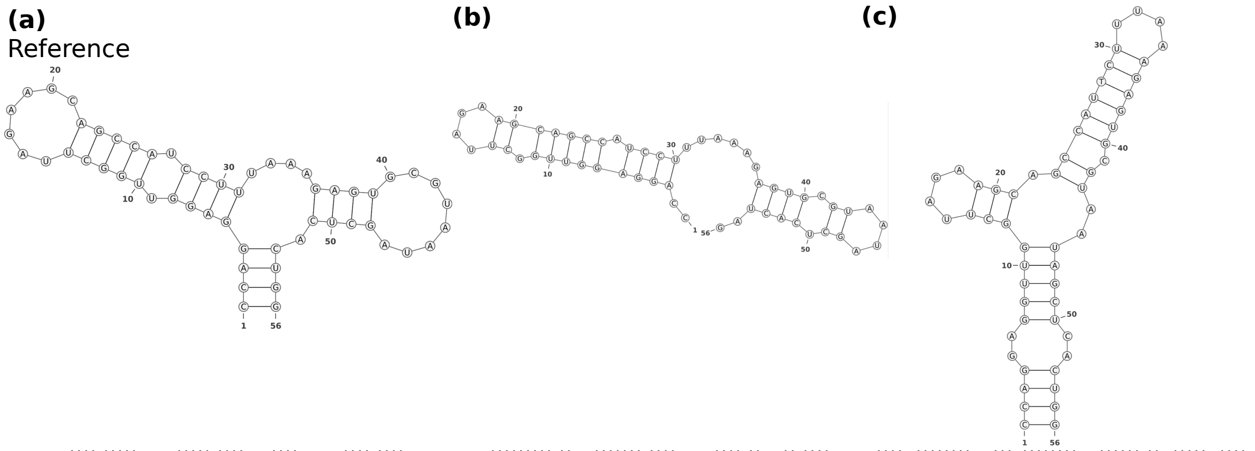

	....((((.(((((.......))))).))))....((((........)))).))))	..(((((((((.((....))))))).))))......((((.((....)).))))..	((((..((((((((....))).((((((((....)))))).))..)))))..))))
Hamming distance		0.32	0.48
RNAdistance "f" (default)		26	44
"h"		32	44
"w"		51	47
"c"		10	9
"F"		16	26
"H"		24	36
"W"		36	43
"C"		6	8
BP distance		34	29
RBP score (t = 1)		3	12
(t = 5)		1	4
(t = 10)		1	3
DoPloCompare		0.16	0.13
AptaMat		6.17	10.65

Figure S6: Graphical representations, dot-bracket notations of the representative structure of ssNA 4 (**a**), and the alternative structures (**b**) and (**c**). The Hamming distance, RNAdistance, BP distance, RBP score using t value within 1, 5 and 10, DoPloCompare, and AptaMat distances from the reference are also reported.

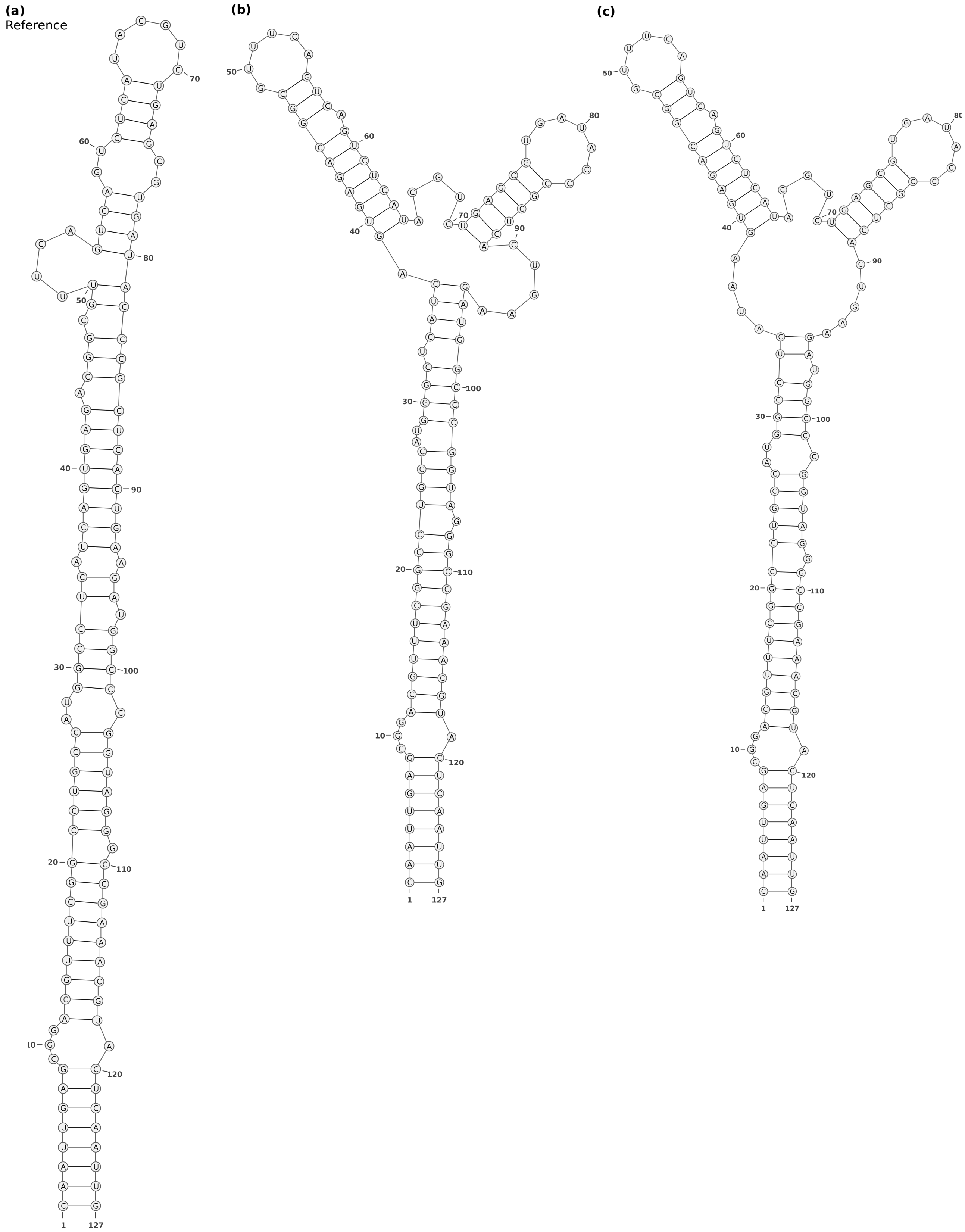

((((((((...((((((((((((((((..((((((.((((((((.(((.((....((((..<br>((((......))))..))))))))))))))))))).)).)))).)))))).))))))))).))))))))	((((((((...((((((((((((((..((((.((((.(((((((((......))).)))<br>))))....(((((((.......)))))).....))))))))))))).))))))))))).))))))))	((((((((...((((((((((((((..((((((....((((((((((......))).))))<br>)))....((((((.......))))))....)).)))).))))).))))))))).))))))))
Hamming distance	0.31	0.29
RNAdistance "f" (default)	60	62
"h"	74	66
"w"	80	79
"c"	14	14
"F"	38	39
"H"	58	67
"W"	74	133.5
"C"	12.5	12
BP distance	55	39
RBP score (t = 1)	14	14
(t = 5)	4	4
(t = 10)	2	2
DoPloCompare	0.023	0.022
AptaMat	8.41	8.65

Figure S7: Graphical representations, dot-bracket notations of the representative structure of ssNA 5 **(a)**, and the alternative structures **(b)** and **(c)**. The Hamming distance, RNAdistance, BP distance, RBP score using t value within 1, 5 and 10, DoPloCompare, and AptaMat distances from the reference are also reported.

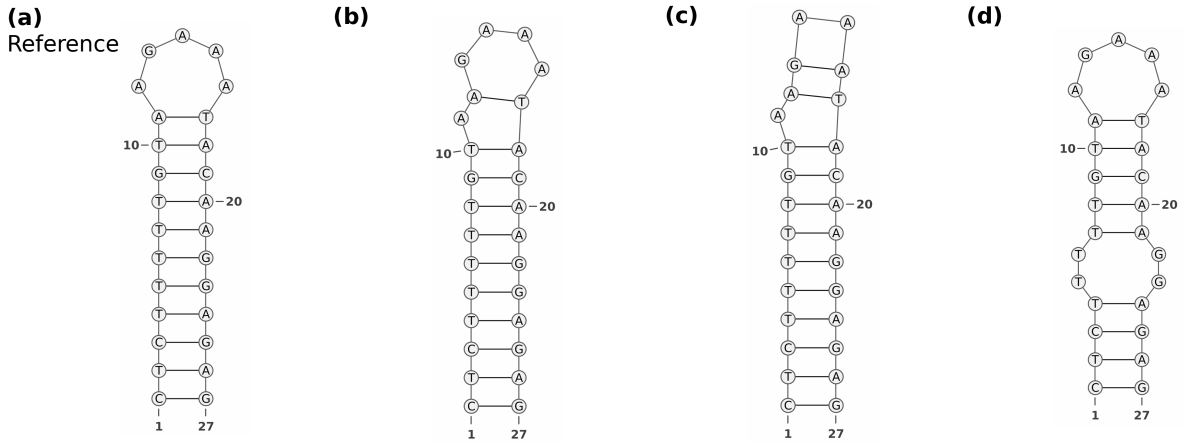

	(a)	(b)	(c)	(d)
	(((((((((((.....)))))))))))	((((((((((.(....)))))))))))	((((((((((.((..))))))))))))	((((..(((((.....)))))..))))
Hamming distance		0.074	0.15	0.15
RNAdistance "f" (default)		2	6	8
"h"		6	10	24
"w"		6	11	18
"c"		3	3	3
"F"		2	4	4
"H"		24	24	24
"W"		15	18	18
"C"		3	3	3
BP distance		2	3	2
RBP score (t = 1, 5, 10)		1	1	1
AptaMat		0.091	0.22	0.20

Figure S8: Graphical representations, dot-bracket notations of the representative structure of ssNA 6 (**a**), and the alternative structures (**b**) and (**c**). The Hamming distance, RNAdistance, BP distance, RBP score using t value within 1, 5 and 10, and AptaMat distances from the reference are also reported.

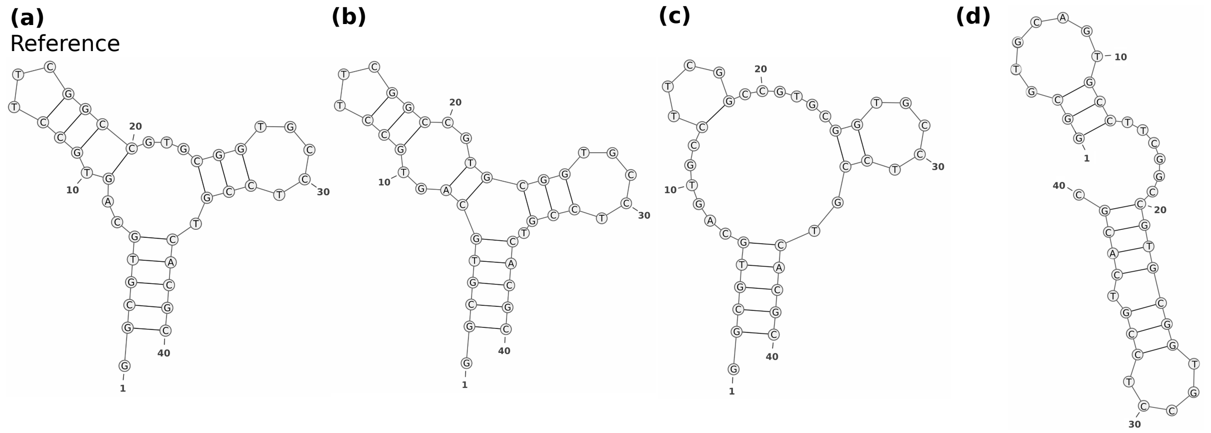

	(a)	(b)	(c)	(d)
	.(((((..(.(((...))))...(((.....))).)))))	.(((((((..(((...)))..))(((.....))).)))))	.(((((......(....)......((.....))..)))))	(((.......)))......(((((((.....))).)))).
Hamming distance		0.15	0.20	0.40
RNAdistance "f" (default)		8	16	32
"h"		8	18	32
"w"		18	24	40
"c"		1	3	9
"F"		6	8	15
"H"		8	18	24
"W"		18	78	35
"C"		1	3	7
BP distance		3	6	16
RBP score (t = 1)		2	2	8
(t = 5)		1	1	3
(t = 10)		1	1	2
AptaMat		0.44	0.70	9.95

Figure S9: Graphical representations, dot-bracket notations of the representative structure of ssNA 7 (**a**), and the alternative structures (**b**) and (**c**). The Hamming distance, RNAdistance, BP distance, RBP score using t value within 1, 5 and 10, and AptaMat distances from the reference are also reported.

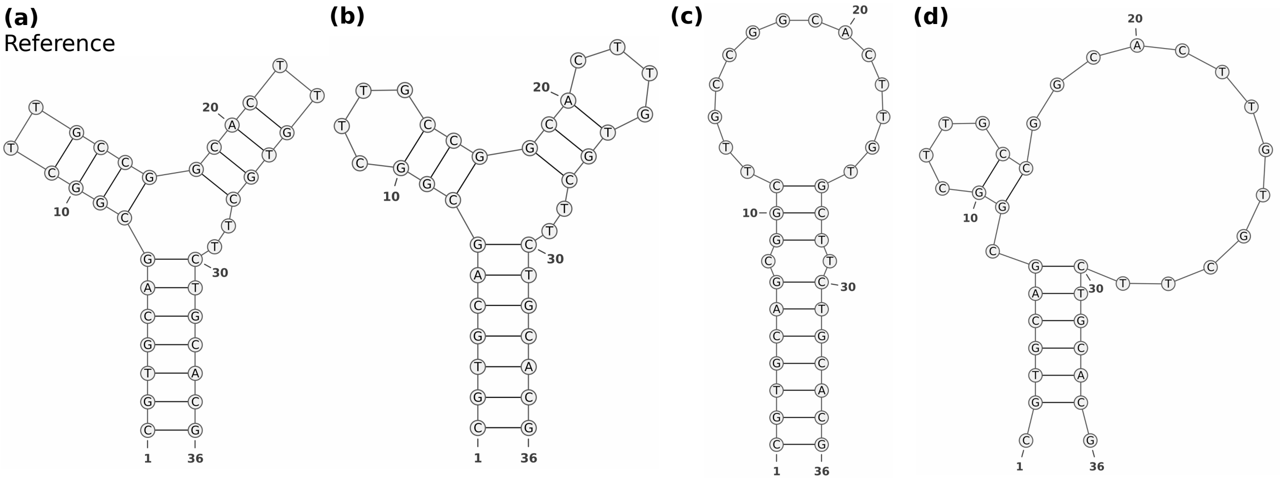

(((((((((((..))))((((..))))..)))))))	((((((((((....)))(((....)))..)))))))	(((((((.(((..............))).)))))))	.((((((.((....)).............)))))).
Hamming distance	0.11	0.33	0.39
RNAdistance "f" (default)	8	24	28
"h"	8	24	28
"w"	10	37	45
"c"	0	5	5
"F"	4	12	14
"H"	8	51	46
"W"	10	91	99
"C"	0	5	5
BP distance	2	11	11
RBP score (t = 1)	1	7	4
(t = 5)	1	2	2
(t = 10)	1	2	2
AptaMat	0.14	4.16	3.21

Figure S10: Graphical representations, dot-bracket notations of the representative structure of ssNA 8 (**a**) and the alternative structures (**b**), (**c**), and (**d**). The Hamming distance, RNAdistance, BP distance, RBP score using t value within 1, 5 and 10 and AptaMat distances from the reference are also reported.

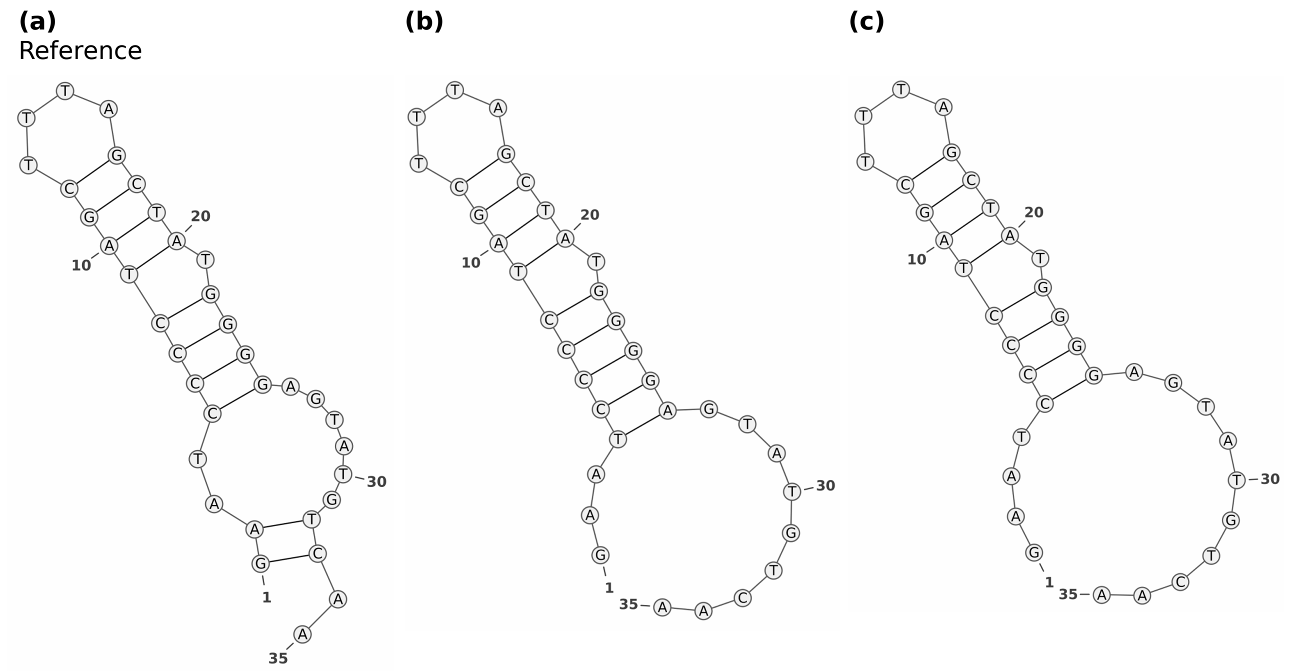

	((..(((((((....)))).))))......))..	...((((((((....)))).))))).........	....(((((((....)))).))))..........
Hamming distance	0.17	0.11
RNAdistance "f" (default)	8	8
"h"	12	12
"w"	29	30
"c"	3	3
"F"	6	4
"H"	12	13
"W"	38	57
"C"	3	3
BP distance	3	2
RBP score (t = 1, 5)	2	2
(t = 10)	1	1
AptaMat	1.05	1.22

Figure S11: Graphical representations, dot-bracket notations of the representative structure of ssNA 9 (**a**) and the alternative structures (**b**) and (**c**). The Hamming distance, RNAdistance, BP distance, RBP score using t value within 1, 5 and 10, and AptaMat distances from the reference are also reported.

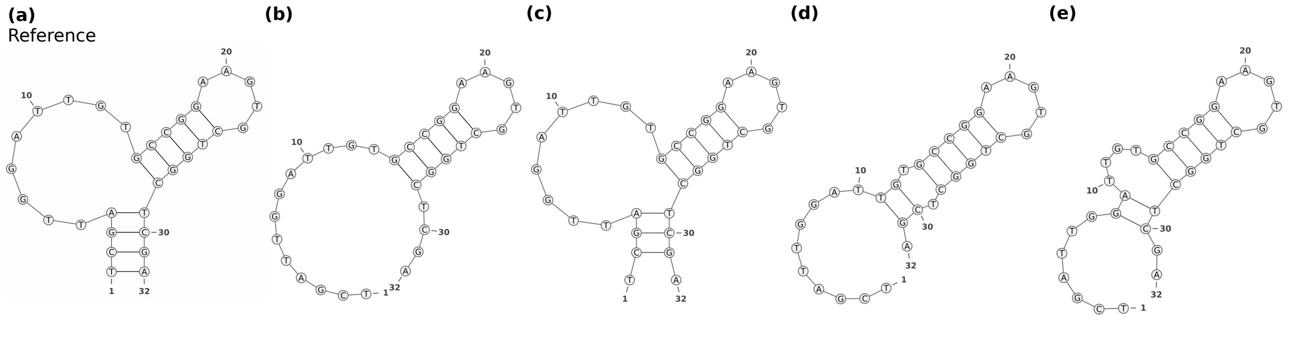

	((((.........(((((.....)))))))))	............(((((.....)))))....	.(((.........(((((.....)))))))).	.........((.(((((.....))))).)).	......((....(((((.....)))))))..
Hamming distance	0.25	0.06	0.25	0.25
RNAdistance "f" (default)	16	4	16	16
"h"	16	4	16	18
"w"	39	3	29	21
"c"	3	0	1	0
"F"	8	2	8	8
"H"	30	4	15	16
"W"	93	3	29	21
"C"	3	0	1	0
BP distance	4	1	6	6
RBP score (t = 1)	4	1	4	4
(t = 5)	2	1	2	2
(t = 10)	2	1	1	1
AptaMat	4	0.12	3	2.25

Figure S12: Graphical representations, dot-bracket notations of the representative structure of ssNA 10 (**a**) and the alternative structures (**b**) and (**c**). The Hamming distance, RNAdistance, BP distance, RBP score using t value within 1, 5 and 10, and AptaMat distances from the reference are also reported.

References

[Berman *et al.*, 2000] Berman, Helen M. and Westbrook, John and Feng, Zukang and Gilliland, Gary and Bhat, T. N. and Weissig, Helge and Shindyalov, Ilya N. and Bourne, Philip E. (2000). The Protein Data Bank. *Nucleic Acids Res*, **28**(3), 235-242.

[Ivry *et al.*, 2009] Ivry, T., and Michal,S., Avihoo,A., Sapiro, G., Barash, D. (2009) An image processing approach to computing distances between RNA secondary structures dot plots., *Algorithms for Molecular Biology*, **4**, 4.

[Kalvari *et al.*, 2021] Kalvari, I., Nawrocki, Eric P., Ontiveros-Palacios, N., Argasinska, J., Lamkiewicz, K., Marz, M., Griffiths-Jones, S., Toffano-Nioche, C., Gautheret, D., Weinberg, Z., Rivas, E., Eddy, Sean R., Finn, Robert D., Bateman, A., Petrov, Anton I. (2021). Rfam 14: expanded coverage of metagenomic, viral and microRNA families. *Oxford Academic*, **49**(D1), 192-200.
